# BMPQ-1 binds selectively to (3+1) hybrid topologies in human telomeric G-quadruplex multimers

**DOI:** 10.1093/nar/gkaa870

**Published:** 2020-10-20

**Authors:** Chao Gao, Zhu Liu, Haitao Hou, Jieqin Ding, Xin Chen, Congbao Xie, Zibing Song, Zhe Hu, Mingqian Feng, Hany I Mohamed, Shengzhen Xu, Gary N Parkinson, Shozeb Haider, Dengguo Wei

**Affiliations:** State Key Laboratory of Agricultural Microbiology, College of Veterinary Medicine, Huazhong Agricultural University, Wuhan 430070, China; National Reference Laboratory of Veterinary Drug Residues (HZAU) and MAO Key Laboratory for Detection of Veterinary Drug Residues, Huazhong AgriculturalUniversity, Wuhan, 430070, China; College of Plant Science and Technology, Huazhong Agricultural University, Wuhan 430070, China; National Key Laboratory of Crop Genetic Improvement, College of Life Science and Technology, Huazhong Agricultural University, Wuhan, 430070, China; State Key Laboratory of Agricultural Microbiology, College of Veterinary Medicine, Huazhong Agricultural University, Wuhan 430070, China; Department of Chemistry, College of Science, Huazhong Agricultural University, Wuhan 430070, China; State Key Laboratory of Agricultural Microbiology, College of Veterinary Medicine, Huazhong Agricultural University, Wuhan 430070, China; Department of Chemistry, College of Science, Huazhong Agricultural University, Wuhan 430070, China; College of Life Science and Technology, Huazhong Agricultural University, Wuhan 430070, China; State Key Laboratory of Agricultural Microbiology, College of Veterinary Medicine, Huazhong Agricultural University, Wuhan 430070, China; Department of Chemistry, College of Science, Huazhong Agricultural University, Wuhan 430070, China; State Key Laboratory of Agricultural Microbiology, College of Veterinary Medicine, Huazhong Agricultural University, Wuhan 430070, China; Department of Chemistry, College of Science, Huazhong Agricultural University, Wuhan 430070, China; State Key Laboratory of Agricultural Microbiology, College of Veterinary Medicine, Huazhong Agricultural University, Wuhan 430070, China; College of Life Science and Technology, Huazhong Agricultural University, Wuhan 430070, China; State Key Laboratory of Agricultural Microbiology, College of Veterinary Medicine, Huazhong Agricultural University, Wuhan 430070, China; Chemistry Department, Faculty of Science, Benha University, Benha 13518, Egypt; Department of Chemistry, College of Science, Huazhong Agricultural University, Wuhan 430070, China; UCL School of Pharmacy, University College London, 29–39 Brunswick Square, London WC1N 1AX, UK; UCL School of Pharmacy, University College London, 29–39 Brunswick Square, London WC1N 1AX, UK; State Key Laboratory of Agricultural Microbiology, College of Veterinary Medicine, Huazhong Agricultural University, Wuhan 430070, China; National Reference Laboratory of Veterinary Drug Residues (HZAU) and MAO Key Laboratory for Detection of Veterinary Drug Residues, Huazhong AgriculturalUniversity, Wuhan, 430070, China

## Abstract

A single G-quadruplex forming sequence from the human telomere can adopt six distinct topologies that are inter-convertible under physiological conditions. This presents challenges to design ligands that show selectivity and specificity towards a particular conformation. Additional complexity is introduced in differentiating multimeric G-quadruplexes over monomeric species, which would be able to form in the single-stranded 3′ ends of telomeres. A few ligands have been reported that bind to dimeric quadruplexes, but their preclinical pharmacological evaluation is limited. Using multidisciplinary approaches, we identified a novel quinoline core ligand, BMPQ-1, which bound to human telomeric G-quadruplex multimers over monomeric G-quadruplexes with high selectivity, and induced the formation of G-quadruplex DNA along with the related DNA damage response at the telomere. BMPQ-1 reduced tumor cell proliferation with an IC_50_ of ∼1.0 μM and decreased tumor growth rate in mouse by half. Biophysical analysis using smFRET identified a mixture of multiple conformations coexisting for dimeric G-quadruplexes in solution. Here, we showed that the titration of BMPQ-1 shifted the conformational ensemble of multimeric G-quadruplexes towards (3+1) hybrid-2 topology, which became more pronounced as further G-quadruplex units are added.

## INTRODUCTION

Four repeats of the human telomeric sequence, (TTAGGG)_*n*_ is able to fold into a monomeric (single unit) G-quadruplex structure (1–2). The central unit of a G-quadruplex is a series of co-planar array of guanines, held together by Hoogsteen hydrogen bonds and stacked on top of another, called the G-quartet stem (3–5). The flanking sequence TTA, contributes to the formation of the loops. X-ray crystallographic and NMR studies have shown that the same sequence can adopt six different topologies including the intramolecular chair, basket, (3+1) hybrid and propeller form (6–9). Experiments have revealed that the sequence can switch between parallel, antiparallel and the hybrid conformations depending upon the precise sequence, metal ions, solvent conditions, oligonucleotide concentration and possibly other factors (10–12). Of these, the 3+1 topology (hybrid-1 and -2) has been demonstrated to be the predominant species in solution (7–9). Very recently, it has also been shown that the human telomeric sequence can form hybrid-type antiparallel G-quadruplex in living human cells (13).

The folding, formation, stabilization and dissolution of the human telomeric G-quadruplexes have been studied by a variety of structural, biophysical and chemical probe methods (14–19). Yet, there is limited data present on the structural arrangements of G-quadruplex long repeats. Most studies reported to date *in vitro* have been on monomeric or dimer telomeric G-quadruplexes, and some extending to not >12 TTAGGG repeats (20–27). However, the 3′ G-rich single strand overhang can be several repeats long *in vivo*, and can form multimeric G-quadruplex units connected by TTA linkers such as the beading model (28–31).

Given the widely distributed G-quadruplex forming sequences in the genome, it has been a challenge to identify novel chemical entities that binds to a particular topology with specificity and selectivity (32–35). Ligands that interact specifically with the telomeric multimeric G-quadruplexes add another layer of complexity to this design process.

Chemical compounds have been shown to stabilize multimeric G-quadruplexes *in vitro* and *in vivo* (36–40). Some complex chemical compounds with large molecular weight such as a zinc-finger-like chiral supramolecular complex (36), di-nickel-salphen linked by (–C–C–O) (26,37,38), and a triaryl-substituted imidazole derivative (39), were identified to bind to dimer G-quadruplex structures, which are constituted by two consecutive quadruplex units (30,40). However, their anti-tumor activity *in vivo* has been scarcely evaluated (23–26,36–38,40). In each of these studies, the dimeric G-quadruplex was assumed form a single, stable conformation under given experiment conditions, and the ligands are considered to interact with the structural unit in a specific conformation (23–26,36–40). Currently, biophysical assays show that a telomeric G-quadruplex forming sequence could fold into multiple conformations in solution (28,29,31). Binding of ligands to telomeric G-quadruplex multimers will involve recognition with multiple conformations in the ensemble. Ligands may interact with the lowest energy conformation, or with one of a number of higher energy conformational substrates that are populated in solution. The ligands may induce a conformational change or lead to a population shift in the G-quadruplex structural ensemble (41,42). The binding affinity need to be described by the free energy change in the whole ensemble instead of the interaction energy calculated from a single complex structure.

**Figure 1.**
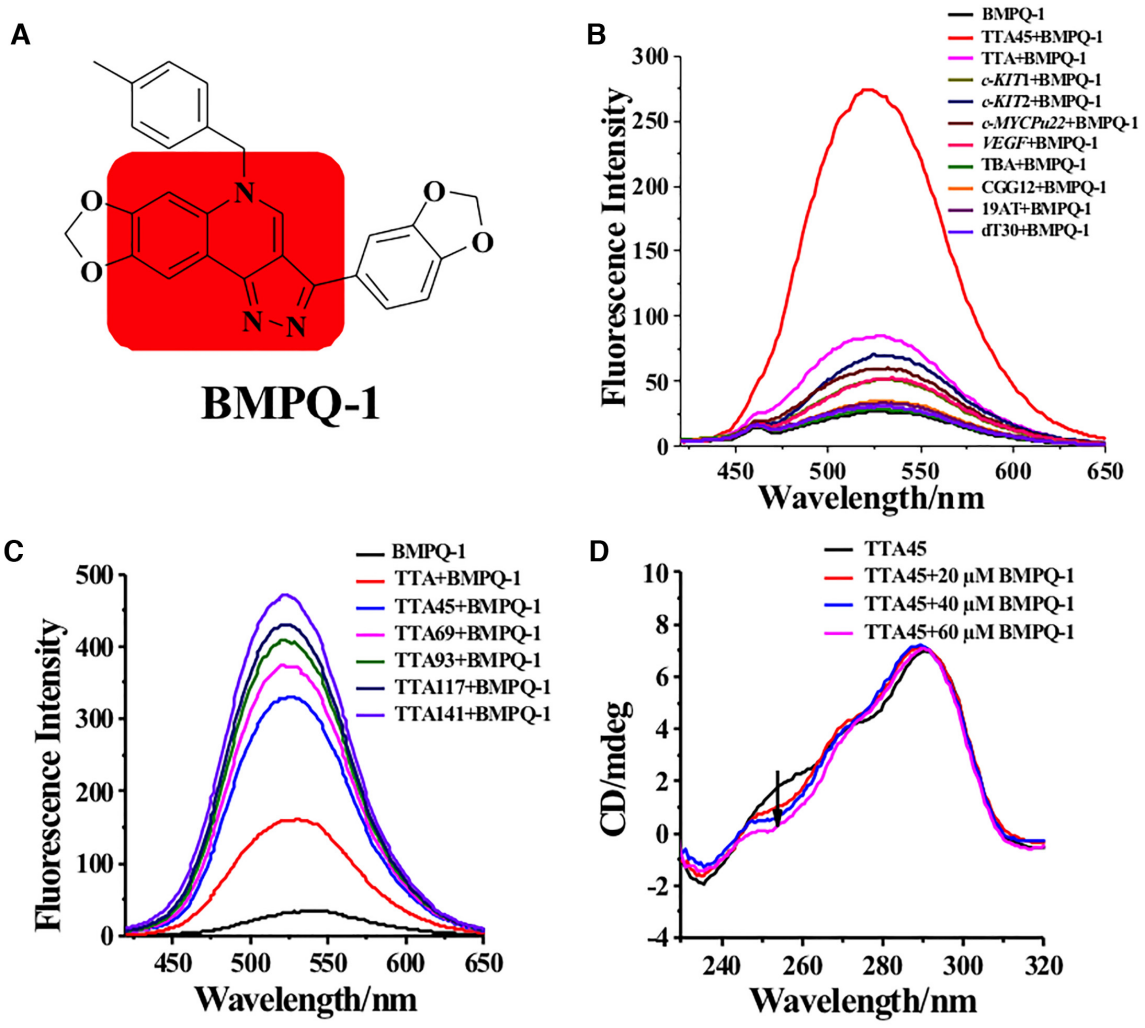
(**A**) Structure of BMPQ-1. (**B**) Fluorescence spectra of BMPQ-1 at λ_ex_ = 395 nm in the absence or presence of dimeric G-quadruplexes (TTA45), monomeric G-quadruplexes (*c-KIT*1, *c-KIT*2, *c-MYCPu22*, TTA, *VEGF*, TBA), trinucleotide DNA (CGG12), duplex DNA (19AT) and single-stranded DNA (dT30). The concentration of BMPQ-1 was 3 μM. The concentration of TTA45 was 2 μM and DNAs with other structures were 4 μM. (**C**) Fluorescence spectra of 6 μM BMPQ-1 at λ_ex_ = 395 nm in the absence or presence of 2 μM telomeric G-quadruplexes. Oligonucleotide samples were prepared in solution with 10 mM K_2_HPO_4_/KH_2_PO_4_, pH 7.0, 100 mM KCl. (**D**) Effect of BMPQ-1 on the CD spectra of 10 μM TTA45 in 10 mM Tris-HCl buffer (pH 7.4) containing 100 mM KCl.

In this study, we report a small chemical compound BMPQ-1 (Figure 1A) with a novel core (5*H*-pyrazolo [4,3-*c*]quinoline) that exhibits selective recognition with multimeric (3+1) hybrid telomeric G-quadruplexes. Immunofluorescence assay showed that BMPQ-1 induced the formation of G-quadruplex DNA at the telomere and the related DNA damage response *ex vivo*. BMPQ-1 inhibited tumor cell proliferation with IC_50_ about 1.0 μM, and decreased the tumor growth rate in mouse by half of the control. At the room temperature, the single molecule fluorescence resonance energy transfer (smFRET) and circular dichroism (CD) studies for the dimeric G-quadruplex (TTA45) indicated that a mixture of multiple conformations coexisted in the solution with potassium. The titration of BMPQ-1 into the dimeric G-quadruplex solution altered the conformational ensemble. This conformational shift was also observed for the recognition that extended to triple, quadruple, quintuple, sextuple G-quadruplex units. In this study, we observe that the change of the conformational distribution in the ensemble plays an important role in the selectivity of BMPQ-1 for multimeric G-quadruplexes. To the best of our knowledge, this is the first example of a ligand shown to selectively bind to (3+1) hybrid multimeric G-quadruplexes and induce conformational changes. This work provides new insights for a ligand targeting multimeric G-quadruplex long repeats.

## MATERIALS AND METHODS

### Materials

All DNA oligonucleotides were synthesized and purified with PAGE by TSINGKE Biological Technology Co., Ltd. (Wuhan, China). The compounds used in the initial screening were from Chinese National Compound library of Peking University (PKU-CNCL). In the following assays, BMPQ-1 and its analogues were purchased from Topscience Bio-Tech Co., Ltd. (Shanghai, China).

### Fluorescence measurements

G-quadruplex forming oligonucleotides were annealed in the buffer (10 mM K_2_HPO_4_/KH_2_PO_4_ pH 7.0, 100 mM KCl) by heating to 95°C for 5 min, followed by gradual cooling to room temperature. Fluorescence spectra were detected by RF-5301PC spectrofluorophotometer (Shimadzu, Japan) at room temperature. The spectra were recorded with 5 nm excitation and emission slit widths. The annealed DNA samples were mixed with compounds at 3 μM in the buffer (10 mM K_2_HPO_4_/KH_2_PO_4_ pH 7.0, 100 mM KCl), and the mixture was incubated for 10 min at 30°C before the spectra collection. For the fluorescence measurements between BMPQ-1 and different DNA sequences, the final concentrations of multimeric G-quadruplex forming oligonucleotides were 2 μM and other DNA oligonucleotides were 4 μM.

For the titration experiments, the concentration of TTA and mutant TTA45 DNA oligonucleotides varied from 0 to 10 μM (0.2, 0.5, 0.7, 1, 1.5, 2, 3, 4, 5, 6, 8, 9, 10 μM), and the concentration of multimeric G-quadruplex forming oligonucleotides (TTA45, TTA69, TTA93, TTA117, TTA141) varied from 0 to 6 μM (0.2, 0.5, 0.7, 1, 1.5, 2, 3, 4, 5, 6 μM). The concentration of BMPQ-1 was 3 μM. After each addition of sample, the reaction was allowed to equilibrate for 10 min at 30°C and fluorescence measurement was taken at λ_ex_ = 395 nm.

### Circular dichroism

CD studies were performed on a Chirascan circular dichroism spectrophotometer (Jasco, Easton, MD, USA). A quartz cuvette with a 1 mm path length was used to record the spectra over a wavelength range of 220–320 nm with a 1 nm bandwidth. All the samples were prepared in buffer containing 10 mM Tris–HCl pH 7.4, 100 mM KCl. To ensure the concentrations of G-quadruplex units to be 20 μM, multiple G-quadruplexes forming oligonucleotides with different concentrations were used (TTA (G1) 20 μM, TTA45 (G2) 10 μM, TTA69 (G3) 6.67 μM, TTA93 (G4) 5 μM, TTA117 (G5) 4 μM, TTA141 (G6) 3.33 μM). The DNA samples were heated at 95°C for 5 min and cooled down slowly to room temperature. 20, 40 and 60 μM BMPQ-1 were mixed with DNAs separately. Melting curves were measured at 295 nm and the range of temperature was 4–95°C.

### Cell cytotoxicity assay

The cell growth effect of BMPQ-1 against different cell lines were tested via measuring the quantity of living cells using the Cell Counting Kit-8 (CCK8) (DOJINDO, Japan) according to the instruction. Cells were seeded in a 96-well plate (5 × 10^3^ cells/well) and grown for 24 h for attachment. Then the cells were treated with various concentrations of BMPQ-1 and incubated at 37°C with 5% CO_2_ for 48 h. After incubation, the old medium was discarded followed by adding fresh medium containing 10% FBS in DMEM. Then 100 μl CCK-8 (10% in grow medium) was added into each well, and the cells were further incubated for 1 h. The optical density (OD) was detected at 450 nm by an automated microplate reader (Bio-Rad). The IC_50_ values were derived from the curves of the mean percentage of survival against the drug concentration. All the experiments were performed in triplicate.

### Cell cycle analysis

A549 cells treated with BMPQ-1 were harvested and washed in PBS and fixed with 70% ethanol at 4°C overnight. Then, the cells were centrifuged and re-suspended in a staining solution (50 μg/mL propidium iodide (PI), 75 KU/ml RNase A in PBS) for 30 min at room temperature in the dark. The cells were analyzed by flow cytometer (BD FacsAria, USA). For each analysis, 2.0 × 10^4^ events were collected.

### Annexin V-FITC/PI apoptosis detection

The A549 cells treated with BMPQ-1 or DMSO were washed twice with PBS and detected using the annexin V-FITC/PI apoptosis detection kit (DOJINDO, Japan). The cells were pelleted and resuspended in the binding buffer. Annexin V-FITC and PI were added, and the cells were disturbed by gently vortexing the samples prior to incubation for 5 min in the dark. Emitted florescence was quantitated by flow cytometry (BD FacsAria, USA). For each analysis, 2.0 × 10^4^ events were collected.

### Confocal imaging of colocalization

The cells (5 × 10^4^ cells) were seeded on 35-mm glass-bottomed dishes and grown for 24 h for attachment. Then old medium was replaced by fresh DMEM containing 1.5 μM BMPQ-1 and incubated for 24 h. After washed three times with PBS buffer, the cells were fixed in 4% paraformaldehyde/PBS for 15 min and then permeabilized with 0.3% Triton-X 100/PBS at 37°C for 20 min. Then the cells were blocked with 5% BSA/PBS at 37°C for 1 h. Finally, the cells were incubated with primary antibodies at 37°C for 3 h. After that, the cells were washed six times with blocking buffer, and were then incubated with secondary antibodies at 37°C for 3 h. Finally, the cells were washed six times with PBS buffer again. The digital images were recorded using a confocal laser scanning microscopy (Olympus FV1000) and analyzed with ImageJ software. One hundred nuclei were counted in each group.

For G-quadruplexes detection, immunofluorescence was performed using standard methods with BG4((PET-28a modified to contain FLAG epitope together and His6 tag), anti-FLAG (primary antibody, 14793S, Cell Signaling Technology) and anti-rabbit Alexa 488-conjugated (secondary antibody, A21206, Life Technology) antibodies). For γH2AX detection, anti-γH2AX (primary antibody, 9718S, Cell Signaling Technology) and anti-rabbit Alexa 488-conjugated (secondary antibody, A21206, Life Technology) antibodies were used. For the detection of TRF2, anti-TRF2 (primary antibody, ab13579, Abcam) and anti-mouse Alexa 555-conjugated (A21427, Life Technology) antibodies were used.

### Xenograft animal model and drug treatments

Male BALB/C-nu/nu mice (4 weeks old) were purchased from the Beijing Vital River Laboratory Animal Technology Co., Ltd. and housed at Experimental Animal Center of Huazhong Agriculture University (Wuhan, China) and maintained in pathogen-free conditions (12 h light–dark cycle at 24 ± 1°C with 60−70% humidity and provided with food and water ad libitum). HT29 cells were harvested during log-phase growth and were resuspended in FBS-free DMEM medium to the density of 4 × 10^6^ cells per 100 μl. 100 μl cells were injected subcutaneously in the right back of the mice. Tumor growth was examined three times a week after implantation, until the tumor volume reached ∼40 mm^3^. The mice were randomly divided into two groups, and each group had five mice. Subcutaneous injection was carried out with BMPQ-1 at a dose of 15 mg/kg for the mice in the experimental group. Mice in the control group were treated with an equivalent volume of normal saline. The volume of the tumor mass was measured with an electronic caliper and calculated as 1/2 × length × width^2^ in mm^3^. The tumors size and the body weight of the mice were measured every day after drug treatment, and growth curves were plotted using average tumor volume within each experimental group. After treatment for 3 weeks, tumor tissues were collected and tested for the weight and the volume.

### Single molecule fluorescence resonance energy transfer (smFRET) analysis

5′- FAM and 3′-Cy5 labeled TTA45 oligonucleotides were annealed in the buffer of 10 mM Tris–HCl (pH 7.4) and 100 mM KCl, by heating to 95°C for 5 min and slowly cooling down to room temperature. smFRET data collection and analysis were performed as previously described (43). In brief, an A1 confocal microscope (Nikon, Japan) coupled to two picosecond pulsed diode laser heads (LDH-P-C-485B and LDH-P-C-640B, PicoQuant, Germany) and two SPCM-AQRH detectors (Excelitas, Canada) was used for fluorescence excitation and emission detection. A 60 × water immersion objective (WI 60×, NA 1.20, Nikon, Japan) was used for confocal laser microscopy, and the pinhole size was set at ∼100 μm. The laser power before the objective was set at ∼100 μW for the 485-nm laser and ∼35 μW for the 640-nm laser. Donor emission was filtered with an ET550/50 m bandpass (Chroma), and acceptor emission was filtered with an ET700/75 m bandpass (Chroma), before being focused onto the two SPCM-AQRH detectors. A pulsed interleaved excitation (PIE) scheme at a repetition of 32 MHz was employed for data collection.

The smFRET measurements were performed at room temperature in pH 7.4 buffer containing 10 mM Tris–HCl, 100 mM KCl, and 0.01% (v/v) Tween 20, with additional 1 mM ascorbic acid and 1 mM methylviologen for photo bleaching and blinking minimization (44). A concentration of ∼100 pM of TTA45 was used for data collection. BMPQ-1 were prepared in DMSO and were incubated with TTA45 about 5 min prior to each experiment to the final desired concentrations. The smFRET data were typically collected for about 1–2 h. Photon time traces were binned with a 1 ms width, and 4–9 counts/bin were used as the threshold for burst searching. The burst searching process was performed using a handwritten script, and a minimum of 25 total photon counts was defined as a burst event. The exact FRET efficiencies were calculated based on our calibrated parameters for the instrument and fluorophores, and the FRET efficiency distribution was analyzed with a multi-Gaussian mixture using a handwritten script.

### Small-angle X-ray scattering analysis

SAXS data were collected at the BL19U2 beamline of the Shanghai Synchrotron Radiation Facility (SSRF) at room temperature. 100 μM annealed TTA45 were prepared in 10 mM K_2_HPO_4_/KH_2_PO_4_, pH 7.0, 100 mM KCl without or with 400 μM BMPQ-1 for SAXS measurement. For each measurement, 20 consecutive frames of 1 s exposure were recorded and averaged, providing no difference between the first and the last frames. The background scattering was recorded for the matching buffer and was subtracted from the TTA45 scattering data. The data was visualized and analyzed using the software package ATSAS (45). The paired-distance *P*(*r*) distribution profiles were obtained by indirect Fourier transformation of the *I*(*q*) scattering profiles using software GNOM.

## RESULTS

### Compounds with 5H-pyrazolo [4,3-*c*] quinoline exhibiting high selectivity for the multimeric G-quadruplex

Based on the conventional G-quadruplex ligand design (46), ∼400 compounds were selected from Chinese National Compound library of Peking University (PKU-CNCL). These compounds were evaluated for their ability to bind to TTA45 by Fluorescence Resonance Energy Transfer (FRET) assay (Supplementary Tables S1 and S2) (47). Compounds 21–26, which contained the 5*H*-pyrazolo [4,3-*c*] quinoline core, exhibited potential to stabilize TTA45 (Supplementary Table S2 and Supplementary Figure S1). Compounds 21–38 were purchased to study their binding with TTA45 by FRET (Supplementary Table S3). Of these, compound 21 (BMPQ-1) displayed the strongest stabilization of TTA45 with a Δ*T*_m_ value 16.1°C in the FRET assay.

The stabilization of TTA45 and TTA by compounds 21–26 (BMPQ-1–BMPQ-6) was further validated by CD melting (Table 1, Supplementary Table S4, Supplementary Figures S2–S4). All the compounds in this series exhibited higher Δ*T*_m_ with TTA45 than with TTA. BMPQ-1 (Table 1) exhibited the highest Δ*T_m_* value with TTA45 of 14.6°C in the CD melting assays, which is much higher than those for the monomer G-quadruplexes (TTA, *c-KIT*1 and TBA G-quadruplex).

**Table 1.** DNA stabilization by BMPQ-1

		Δ*T*_m_ (°C)^a^					
Compound	Structure	TTA	TTA45	TTA69	TBA	*c-KIT1*	Duplex
BMPQ-1	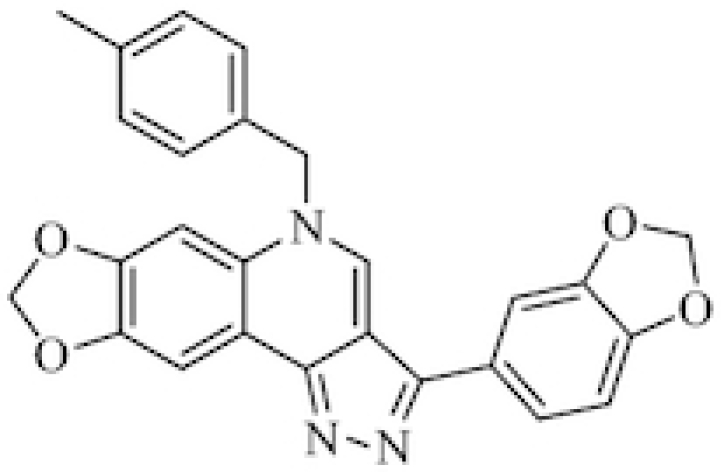	8.4	14.6	13.8	4.7	6.6	1.8

^a^Melting temperature (*T*_m_) of 5 μM DNA samples (Table 1 and S1) with and without 20 μM BMPQ-1 in 10 mM

Tris–HCl buffer (50 mM KCl, pH 7.4) was determined by circular dichroism spectroscopy, and Δ*T*_m_ (°C) were derived by their difference.

A comparison of structure-activity relationships (SAR) indicated that the 1,3-dioxolane ring at positions R1 and R2 (Supplementary Table S3) contributed to higher greater stabilization of TTA45. A lack of the dioxolane ring in compounds 28 and 29 resulted in lower stabilization of TTA45. However, an increase in ring size does not further affect compound's ability to stabilize TTA45 (compound 33 and BMPQ-5). Compounds 37 and 38 with phenyl at position R4 exhibited negligible stabilization of TTA45. This suggested that the substitutions at 4*H*-pyrazole could interfere with the interactions. The difference between the stabilization ability of BMPQ-4 and those of BMPQ-1 and BMPQ-2 highlighted that the phenyl in 1,4-dihydropyridine at R3 performed better than the aliphatic chains (Supplementary Table S3). This suggested that a π–π stacking might exist between R3 and TTA45.

### Fluorescence selectivity of BMPQ-1 for the multimeric G-quadruplexes *in vitro*

Fluorescence response to different DNA structures was employed to validate the selectivity of BMPQ-1 for multimeric G-quadruplexes. With the addition of dimeric G-quadruplexes to BMPQ-1, the fluorescence at 521 nm increased 10-fold (Figure 1B). In contrast, the fluorescence enhancements were less than 3-fold when BMPQ-1 was mixed or titrated with other monomeric DNA G-quadruplex sequences, including anti-parallel G-quadruplex (TBA), parallel G-quadruplex (*c-KIT*1, *c-KIT*2, *c-MYC Pu22*, *VEGF*), mixed G-quadruplex (TTA), duplex (19AT), single strand (dT30) (Figure 1B, Supplementary Table S1). The quantum yield variance of BMPQ-1 with these G-quadruplexes is illustrated in Supplementary Figure S5. The fluorescence intensity of BMPQ-1 increased with the titration of multimeric G-quadruplexes with successive G-quadruplex units being added to the nucleotide sequence (Figure 1C and Supplementary Figure S6). This suggested that BMPQ-1 might exhibit stronger selectivity for the guanine rich sequence at the telomeres in cells.

The structure of TTA45 was assumed to fold into a dimeric G-quadruplex linked by a TTA loop. Three mutant telomeric DNA sequences (TTA43, TTA51/T3 and TTA57/T5) with loops of different lengths were designed to study the effect of the loop length on the binding (Supplementary Table S1). Comparing with the mutants (TTA43, TTA51/T3 and TTA57/T5) at the same concentration, TTA45 enabled BMPQ-1 to emit the strongest fluorescence (Supplementary Figure S7). This suggests that the TTA loop in TTA45 is optimal for binding. The mutant (A-TTA45-A) with two extended A on both sides of TTA45 makes BMPQ-1 exhibit weaker fluorescence. This indicated that an extra A could interfere the recognition between BMPQ-1 and TTA45. The slower fluorescence intensity increase of BMPQ-1 with the titration of mutants (Supplementary Figure S8) further proved the assumption.

### DNA damage response triggered by BMPQ-1 at the telomeres

The *ex vivo* results showed that BMPQ-1 stabilized telomeric multimeric G-quadruplexes. This encouraged us to further evaluate its ability to disrupt telomere maintenance. The stabilization of the telomeric G-quadruplexes by ligands could alter the T-loop structure in the chromosome ends, and the unprotected chromosome ends could be recognized and repaired as double-strand breaks (DSBs) to trigger DNA damage responses. Moreover, uncapped telomeres would experience degradation, inappropriate recombination, and chromosomal end-to-end fusion events. The telomere dysfunction coming from the G-quadruplex ligands can lead to phenomena similar to that of DNA damage in eliciting cell cycle arrest and apoptosis (48).

**Figure 2.**
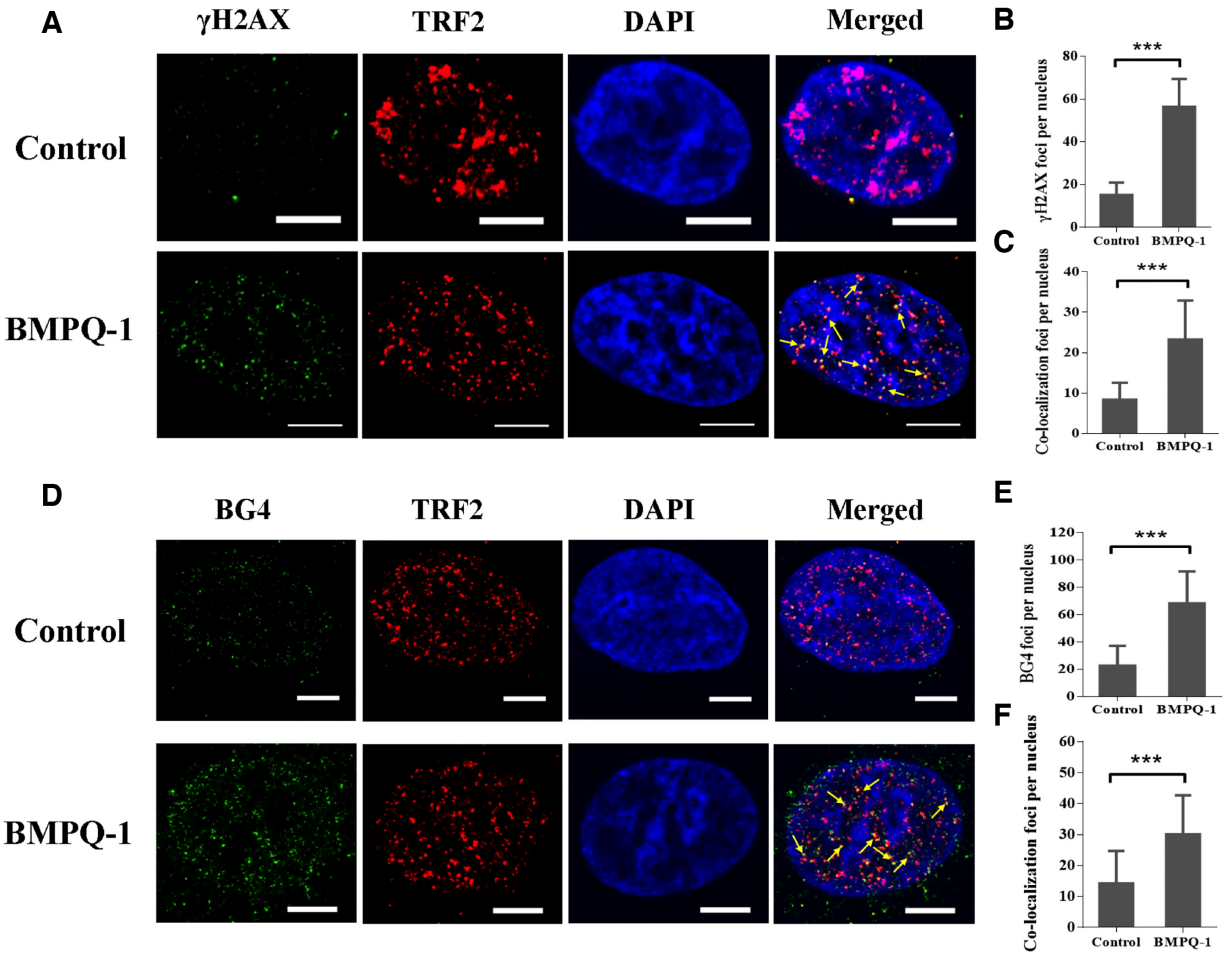
(**A**) Representative immunofluorescence images of γH2AX (green) and TRF2 (red) foci in A549 cells treated without or with 1.5 μM BMPQ-1 for 24 h. The nuclei were stained with DAPI (blue), and typical co-localization foci are indicated by yellow arrows. (**B**) Quantification of the number of γH2AX foci per nucleus in A549 cells treated with or without BMPQ-1. (**C**) Quantification of the colocalization of γH2AX and TRF2 per nucleus in A549 cells treated with or without BMPQ-1. (**D**) Representative immunofluorescence images of BG4 (green) and TRF2 foci in A549 cells treated with or without 1.5 μM BMPQ-1 for 24 h. (**E**) Quantification of the number of BG4 foci per nucleus in A549 cells treated with or without BMPQ-1. (**F**) Quantification of the colocalized BG4 and TRF2 per nucleus in A549 cells treated with or without BMPQ-1. Scale bar = 5 μm. ***P < 0.001, significantly different from the control.

Immunofluorescence experiments were employed to investigate the DNA damage response induced by BMPQ-1 (Figure 2A), which could be characterized by the increase of histone H2AX (γH2AX, a hallmark of DNA damage) foci (48–50). The telomeric repeat binding factors 2 (TRF2) antibody, a specific antibody against the telomere binding protein TRF2, is widely used to locate telomeres (51–53). The co-localization between γH2AX foci and TRF2 could be employed to show that the DNA damage occurred at the telomeric region (Figure 2A). Compared with the control, the treatment with BMPQ-1 trebled the average number of γH2AX foci in individual cells, and the increase on the co-localization between γH2AX foci and TRF2 was observed with statistical significance (Figure 2B, C). This clearly showed the damage induced by BMPQ-1 at the telomeric region.

### Induction of the formation of telomeric G-Quadruplex DNA by BMPQ-1 *in vivo*

Immunofluorescence assay with the BG4 antibody was employed to quantify and visualize DNA G-quadruplexes in human cells treated with BMPQ-1 (54,55). As shown in Figure 2D and E, the treatment of BMPQ-1 for 24 h induced a significant increase on the amount of the BG4 foci in the nucleus. To further check whether the increase of BG4 foci was located at the telomere region, dual-colour immunofluorescence experiments using BG4 and the telomeric repeat binding factors 2 (TRF2) antibody, a specific antibody against telomere binding protein TRF2, were performed to visualize the G-quadruplex and telomeres, respectively. After the treatment with BMPQ-1 for 24 h, the amount of the co-localized BG4 and TRF2 protein foci significantly increased (Figure 2D–F), suggesting the induction of endogenous telomeric G-quadruplex structures in cells by BMPQ-1.

### BMPQ-1 induces cell cycle arrest and apoptosis

Flow cytometry assays were carried out to check the effect of BMPQ-1 on cancer cell cycle arrest or apoptosis. Propidium iodide (PI) in conjunction with Annexin V was used to determine if cells were viable, apoptotic, or necrotic through differences in plasma membrane integrity and permeability (56). Following treatment by 3 μM BMPQ-1 for 24 h, the percentage of G2 / M phase in A549 cells increased from 10.4% to 17.8% (Supplementary Figure S9), and the population of apoptotic cells reached approximately 60% (Supplementary Figure S10). This indicated the effect of BMPQ-1 on inducing apoptosis.

### Effects of BMPQ-1 on tumor cell proliferation

**Figure 3.**
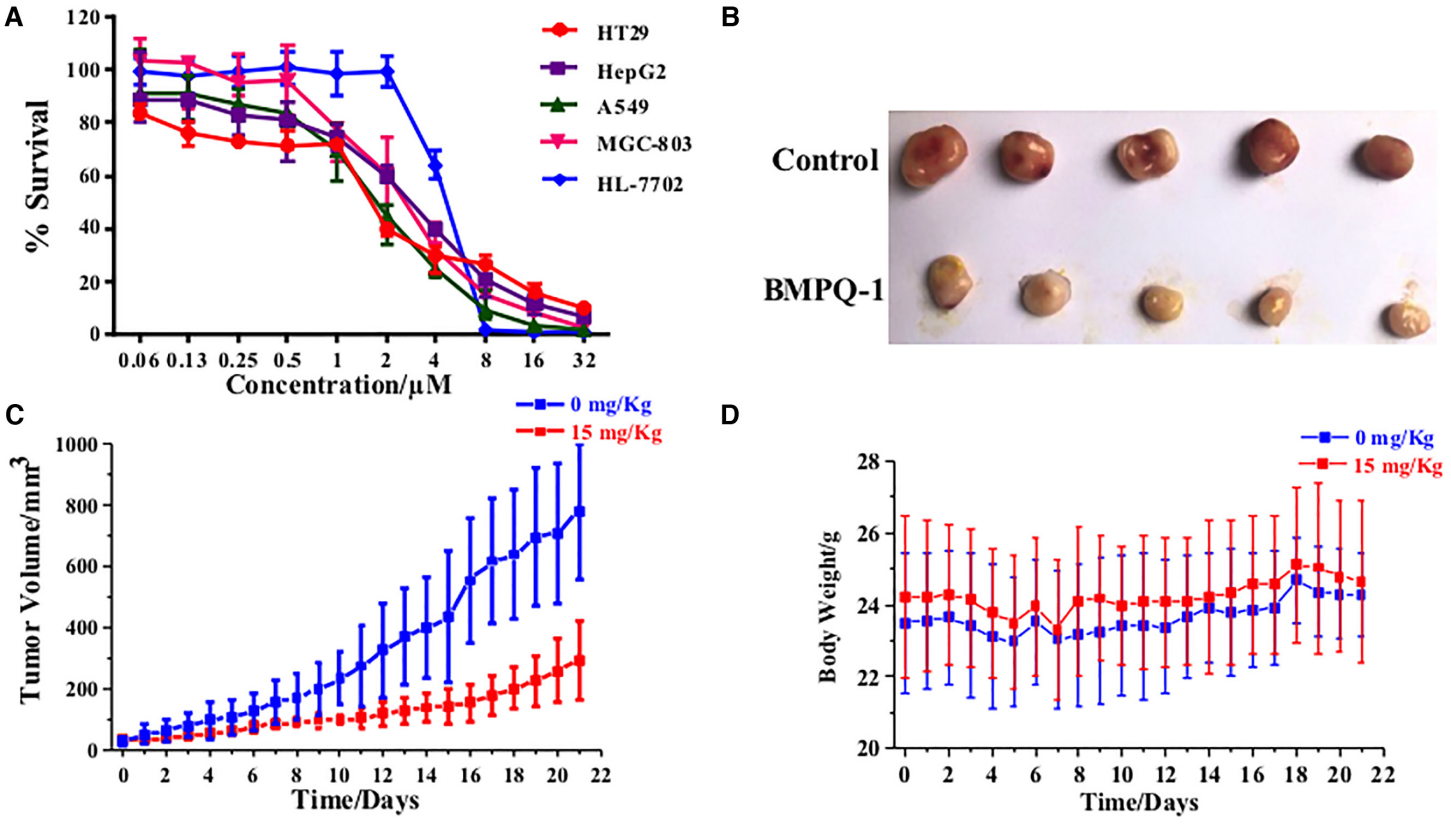
(**A**) Cell growth inhibition curves of tumor cells and normal cells after 48 h treatment with BMPQ-1. The data is reported as the percentage of growing cells treated with BMPQ-1 when compared with that of the untreated cells. (**B**) Excised tumors from each group. (**C**) BMPQ-1 inhibited the tumor growth in a HT29 xenograft model *in vivo*. Tumor volumes were measured daily. (**D**) Body weight of the mice in each group during the period. The data were represented as the mean ± SD.

A Cell Counting Kit-8 (CCK8) (DOJINDO, Japan) was employed to evaluate the cytotoxicity of BMPQ-1 on human alveolar basal epithelial cancer cell line (A549), human hepatocellular carcinoma cancer cell line (HepG2), human gastric cancer cell line (MGC-803), human colon adenocarcinoma (HT29), human normal liver Cells (HL-7702). BMPQ-1 led to a significant dose-dependent cytotoxic effect on cancer cell line with IC_50_ values 1.40 ± 0.06 μM (HT29), 1.46 ± 0.16 μM (A549), 2.14 ± 0.26 μM (HepG2), 2.36 ± 0.48 μM (MGC-803), respectively (Figure 3A). BMPQ-1 also induced growth inhibition in human normal liver cells (HL-7702) with an IC_50_ of 4.38 ± 0.39 μM. Based on the cell growth inhibition curves, compound BMPQ-1 at 2 μM exhibited the highest selectivity for the tumor cells against the normal cells (HL-7702).

### Inhibition of tumor growth by BMPQ-1 *in vivo*

BMPQ-1 exhibited strong toxicity towards cancer cells, which prompted us to investigate its antitumor potential. We tested BMPQ-1 in a HT29 xenograft mouse model of human colon adenocarcinoma. HT29 tumors were established through the subcutaneous injection of HT29 cells into the right back of BALB/C-nu/nu mice (57,58). The mice were divided into normal saline treated group, and BMPQ-1 (15 mg/kg) treated group, *n* = 5/group. Subcutaneous injection was given daily. After 3 weeks treatment, the tumors were excised and measured for final size (Figure 3B). The tumor volume in the normal saline group reached 700 mm^3^ (Figure 3C). In contrast, in the tumor-bearing mice treated with BMPQ-1, the tumor volume was ∼300 mm^3^. The tumor weight also exhibited an obvious decrease in BMPQ-1 treated group (Supplementary Figure S11). However, no significant change was observed in the mouse body weight after the treatment with BMPQ-1 (Figure 3D). These results demonstrated BMPQ-1 effectively inhibited the tumor growth *in vivo*.

### Effect of BMPQ-1 on the CD spectra of the telomeric G-quadruplex multimers

In the absence of 3D structures of multimeric G-quadruplexes, biophysical methods like CD spectra has been used extensively as an intuitive tool to characterize the conformations of the G-quadruplexes and to analyze the influence of small molecules on their conformations (11,38). The CD spectra of the monomeric and multimeric G-quadruplexes exhibited similar spectroscopic trends, a positive peak at 290 nm, a negative peak at ∼240 nm, and two shoulders at about 250 nm and 270 nm, respectively (Figure 1D and Supplementary Figure S12). This indicated that the monomeric and multimeric G-quadruplexes shared similar conformational distribution in solution. The addition of BMPQ-1 did not change the shoulder at 270 nm, however the 250 nm shoulder decreased with increasing ligand concentrations.

### Single molecule fluorescence resonance energy transfer (smFRET) analysis for the binding of BMPQ-1 with the conformational ensemble of TTA45

**Figure 4.**
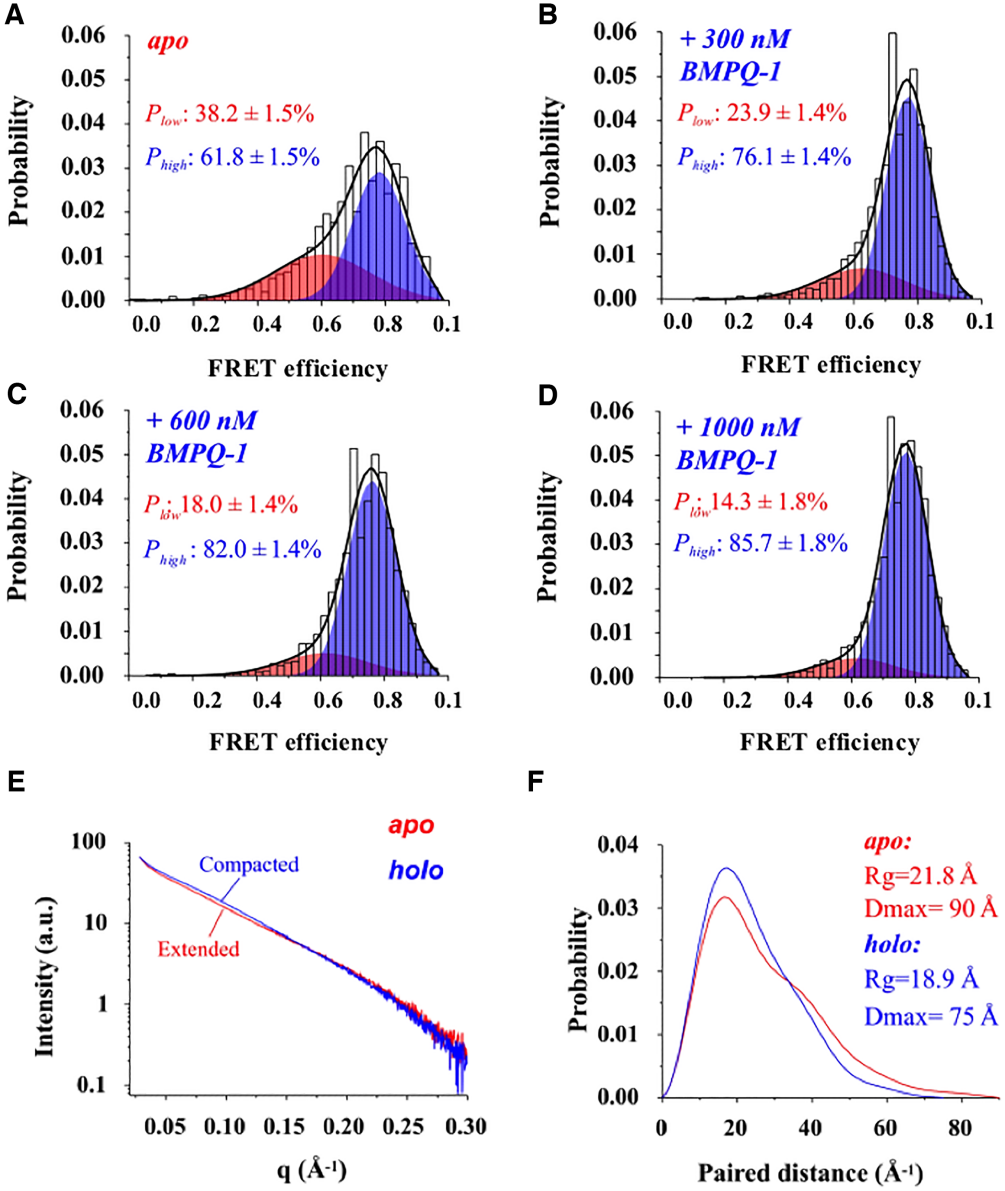
Conformational dynamics of TTA45 and mechanistic analysis of the interaction between TTA45 and BMPQ-1. (**A**) The smFRET profile (grey histogram) of apo TTA45, and it can be fitted as the sum of the two FRET species (black line). The low- and high-FRET species are colored red and blue, respectively. (**B–D**) The high-FRET species of TTA45 is enriched upon the increasing of BMPQ-1. (**E**) SAXS profiles of the apo TTA45 (red line) and the holo TTA45 (blue line) and (**F**) paired-distance distribution curves transformed from the SAXS profiles. The populations of the smFRET species are average over three independent measurements, with the errors indicating 1SD.

To assess the conformational population in solution and reveal the mechanistic insight of BMPQ-1 recognition of TTA45, we performed single-molecule fluorescence resonance energy transfer (smFRET) analysis (59,60). The fluorescent donor FAM and acceptor Cy5 were attached at the 5′ and 3′ end of TTA45 oligonucleotide, respectively. After annealing, the conformation of the dimeric G-quadruplex was characterized using smFRET. It turned out that the FRET profile of TTA45 could be clearly fitted to two FRET species. The low- and high-FRET efficiencies were centered at 0.6 and 0.8, with respective populations of about 38% and 62% (Figure 4A).

To characterize the interaction between BMPQ-1 and TTA45, we titrated BMPQ-1 into TTA45 and monitored the smFRET profile changes. The high-FRET species was enriched with no apparent FRET efficiency shift during BMPQ-1 titration, while the low-FRET population was reduced (Figure 4A–D). By tracing the population changes of high-FRET species against BMPQ-1 concentrations, the dissociation constant (*K*_D_) value was derived as 0.53 μM (Supplementary Figure S13). Taken together, BMPQ-1 selectively recognizes TTA45 via a conformational selection mechanism in which a ligand stabilizes and enriches a pre-existing conformation of a dynamic biomolecule by equilibrium shift mechanisms. The high population is hybrid-2, while the low population can be attributed to hybrid-1, basket or other possible conformations. The conformational ensemble analysis provides new insight towards understanding the recognition between ligands and the G-quadruplex forming sequences.

### Small-angle X-ray scattering (SAXS) analysis to study the effect of BMPQ-1 on the TTA45 conformational ensemble

The conformational changes in TTA45 upon BMPQ-1 binding were also corroborated by small angle X-ray scattering (SAXS) characterization (61). In the absence of BMPQ-1 (*apo* form), the maximum distance (D_max_) and the gyration radius (Rg) of the TTA45 was 90 and 21.8 Å, respectively. Then, in the presence of BMPQ-1 (*holo* form), the *D*_max_ and the *R*_g_ was reduced to 75 and 18.9 Å, respectively. It implies that BMPQ-1 switches the TTA45 structure from an extended state to a more compact state (Figure 4E and F). This is consistent with the smFRET data that BMPQ-1 enriches the TTA45 compact conformation (Figure 4A–D).

## DISCUSSION

The telomere end contains guanine rich sequences, which can form a G-quadruplex cluster consisting of multiple G-quadruplex units. There have been several attempts to search for chemical compounds selectively targeting telomeric G-quadruplex. However, the lack of structural information presents a challenge for understanding the recognition mechanism between the ligand and the telomere multimeric G-quadruplex units. In this work, monomeric and multimeric telomere G-quadruplexes exhibited similar CD spectral characteristics, and with the titration of BMPQ-1, their CD spectra showed similar variations (Figure 1D and Supplementary Figure S12). This suggested that when interacting with ligands, multimeric G-quadruplexes shared similar recognition mechanism as the single G-quadruplex. For this reason, with the titration of BMPQ-1, conformational change of G-quadruplex monomers was used to speculate the conformational change of multimeric G-quadruplexes.

Several G-quadruplex monomer structures have been resolved by X-ray crystallography and NMR. Generally, the G-quadruplex forming sequences fold into a single G-quadruplex structure in the crystalline form; however, in solution, multiple conformations may coexist. The ratio of different conformations depends on the oligonucleotides at its 5′/3′ ends, cations, chemical compounds in solution, etc. Recently, Yang *et al.* reported that, in solution with potassium, Tel 26 mainly folds into a (3+1) Hybrid-1 G-quadruplex (Supplementary Figure S14A), and Wt-Tel 26 mainly exists in (3+1) Hybrid-2 (8,10,22). In the CD spectrum of Wt-Tel 26 in potassium solution, there are a negative peak at about 240 nm, a positive peak at ∼290 nm, and a shoulder peak at about 270 nm (Supplementary Figure S14B). These three peaks match well with a part of the CD spectra of multimeric G-quadruplex forming oligonucleotides in solution with potassium (Figure 1D and Supplementary Figure S12). In the CD spectrum of Tel 22 in solution with potassium, there is a shoulder peak at about 250 nm, which was suggested to come from the co-existence of the hybrid and basket conformations in the solution (6). The shoulder peak at about 250 nm also exists in the CD spectrum of multimeric G-quadruplexes. Therefore, we speculate that in addition to the hybrid G-quadruplexes, some basket G-quadruplexes may exist in the multimeric G-quadruplexes solution. When BMPQ-1 was titrated into the TTA45 solution, the 250 nm shoulder in the CD spectrum decreased, which may come from the distortion of the basket conformation. When BMPQ-1 was titrated into Tel26 solution with potassium, the peak at 265 nm in the CD spectrum decreased (Supplementary Figure S14C), and the newly generated CD spectrum was similar to the CD spectrum of Wt-Tel 26 (Supplementary Figure S14B). This suggested that BMPQ-1 might induce transition between hybrid conformations. To the best of our knowledge, this is the first example of a ligand that selectively binds to multimeric G-quadruplexes by changing the distribution of the ensemble to the (3+1) hybrid conformation.

BMPQ-1 (MW 437.45) selectively binds to (3+1) hybrid conformations and exhibits stronger inhibition against tumor cells than the classic G-quadruplex ligands like BRACO-19 and RHPS4 (Supplementary Figure S15, Supplementary Table S5). The compound alters the conformational equilibrium of telomere multimeric G-quadruplexes in solution in favor of (3+1) conformation, which contributes to its selectivity. Meanwhile, this compound did not have influence on the conformations of other non-telomeric G-quadruplexes (Supplementary Figure S16). Both the ligand and the low energy barrier between the switchable conformations contribute to the conformation ensemble change. This is similar to the principle employed for designing allosteric inhibitors where the druggable compound triggers a switch in the conformational ensemble of a specific receptor and in the processes generates unexpected selectivity towards the receptor. This, we believe will provide interest in the design of the next generation of G-quadruplex interactive compounds.

## Supplementary Material

gkaa870_Supplemental_File
