# Supplementary material for: BMPQ-1 binds selectively to (3+1) hybrid topologies in human telomeric G-quadruplex multimers
Source: Nucleic Acids Res. 2020 Oct 20;48(20):11259–69. doi: 10.1093/nar/gkaa870 (PMC7672424; doi:10.1093/nar/gkaa870)
Supplement: gkaa870_Supplemental_File [file gkaa870_supplemental_file.pdf]

Supporting Information

BMPQ-1 binds selectively to (3+1) hybrid topologies in human telomeric G-quadruplex multimers

Chao Gao [1,2,3]†, Zhu Liu [4]†, Haitao Hou [1,5]†, Jieqin Ding [1,5], Xin Chen [6], Congbao Xie [1,5], Zibing Song [1,5], Zhe Hu [1], Mingqian Feng [6], Hany I Mohamed [1,2,7], Shengzhen Xu [5]*, Gary N Parkinson [8], Shozeb Haider [8]* and Dengguo Wei [1,2]*

[1] State Key Laboratory of Agricultural Microbiology, Huazhong Agricultural University, Wuhan 430070, China

[2] National Reference Laboratory of Veterinary Drug Residues (HZAU) and MAO Key Laboratory for Detection of Veterinary Drug Residues, Huazhong Agricultural University, Wuhan 430070, China

[3] College of Plant Science and Technology, Huazhong Agricultural University, Wuhan 430070, China

[4] National Key Laboratory of Crop Genetic Improvement, College of Life Science and Technology, Huazhong Agricultural University, Wuhan, 430070, China

[5] Department of Chemistry, College of Science, Huazhong Agricultural University, Wuhan 430070, China

[6] College of Life Science and Technology, Huazhong Agricultural University, Wuhan, 430070, China

[7] Chemistry Department, Faculty of Science, Benha University, Benha 13518, Egypt

[8] UCL School of Pharmacy, University College London, 29-39 Brunswick Square, London WC1N 1AX, U.K.

* To whom correspondence should be addressed. Email: dgwei@mail.hzau.edu.cn; xusz@mail.hzau.edu.cn; Shozeb.haider@ucl.ac.uk

† The authors wish it to be known that, in their opinion, the first three authors should be regarded as Joint First Authors.

Table S1. DNA sequences used in the experiment

Name	Type/origin	Structures	Sequences (5'→3')
**DNA G-quadruplex forming oligonucleotide sequences**			
TBA	G4-Aptamer	Antiparallel	GGTTGGTGTGGTTGG
c-MYC Pu22	G4-Promoter	Parallel	TGAGGGTGGGTAGGGTGGGTAA
*c-KIT*1	G4-Promoter	Parallel	AGGGAGGGCGCTGGGAGGAGGG
*c-KIT*2	G4-Promoter	Parallel	GGGCGGGCGCGAGGGAGGGG
VEGF	G4-Promoter	Parallel	GGGAGGGTTGGGGTGGG
TTA	G4-Human telomere	Mixed	GGGTTAGGGTTAGGGTTAGGG
TTA 45	G4-Human telomere	Mixed	$(GGGTTA)_7GGG$
TTA43	Mutant G4	Mixed	$GGG(TTAGGG)_3TGGG(TTAGGG)_3$
TTA51/T3	Mutant G4	Mixed	$GGG(TTAGGG)_3(TTA)_3GGG(TTAGGG)_3$
TTA57/T5	Mutant G4	Mixed	$GGG(TTAGGG)_3(TTA)_5GGG(TTAGGG)_3$
Tel26	G4-Human telomere	Hybrid-1	$AAAGGG(TTAGGG)_3AA$
Wt-Tel26	G4-Human telomere	Hybrid-2	$(TTAGGG)_4TT$
FAM-TTA45 -Cy5	G4-Human telomere	Mixed	FAM-$(GGGTTA)_7$GGG-Cy5
FAM-TTA45 -TAMRA	G4-Human telomere	Mixed	FAM- $(GGGTTA)_7$GGG -TAMRA
**DNA Non-G-quadruplex forming oligonucleotide sequences**			
CGG12	Trinucleotide	Trinucleotide	$(CGG)_{12}$
19AT	Duplex	Duplex	ACGTCGATTATAGACGAGC
19AT-couple		Mixed	GCTCGTCTATAATCGACGT
dT30	Single strand	Single strand	TTTTTTTTTTTTTTTTTTTTTTTTTTTTTT

Table S2. Stabilization of TTA45 as determined by FRET assays.

Compounds	Structures	ΔT_m/°C	Compounds	Structures	ΔT_m/°C
1	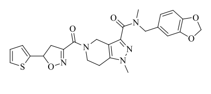	0.70	12	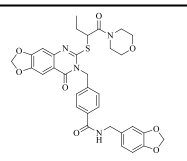	5.11
2	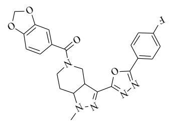	1.90	13	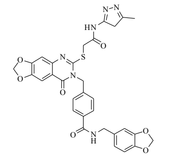	5.67
3	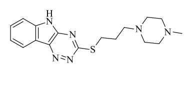	9.04	14	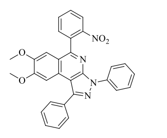	3.85
4	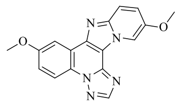	3.22	15	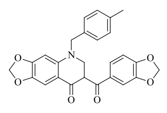	0.70
5	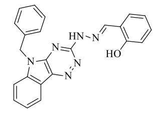	1.53	16	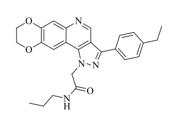	1.12
6	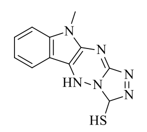	0.28	17	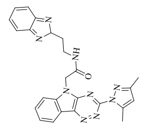	2.17
7	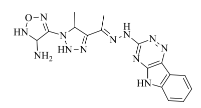	1.33	18	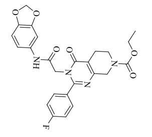	0.06
8	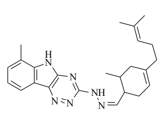	1.61	19	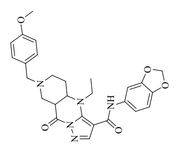	0.56
9	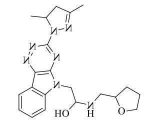	0.63	20	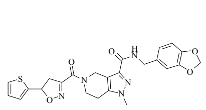	0.70
10	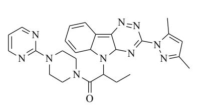	1.05	21 (BMPQ-1)	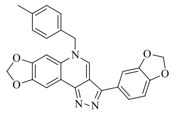	16.1
11	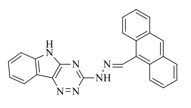	1.54	22 (BMPQ-2)	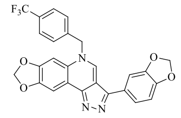	12.6

Table S2. Stabilization of TTA45 as determined by FRET assays (continued).

Compounds	Structures	ΔT_m/°C	Compounds	Structures	ΔT_m/°C
23 (BMPQ-3)	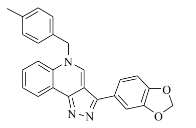	10.3	25 (BMPQ-5)	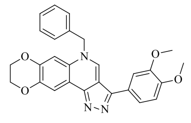	14.6
24 (BMPQ-4)	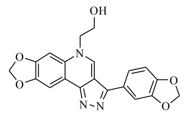	7.9	26 (BMPQ-6)	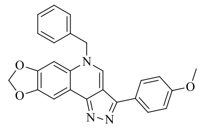	11.7

Melting temperature (T_m) of 0.5 µM TTA45 with and compounds in solution (10 mM K_2HPO_4/KH_2PO_4, 100 mM KCl, pH 7.0) was determined by FRET procedure, and □T_m/°C were derived by their difference. The concentration of compounds 21-26 were 20 µM, and other compounds were 50 µM.

FRET assay was carried out on the Real-Time PCR 3.2 machine (Analytik Jena, Germany). The total reaction volume was 20 µL with 0.5 µM of oligonucleotide labelled with FAM at the 3' end and TAMRA at 5' end in 10 mM K_2HPO_4/KH_2PO_4 (pH 7.0) containing 100 mM KCl. The DNA samples were heated at 95 °C for 5 min, followed by gradual cooling to room temperature. Then compounds with various concentrations were added into the DNA samples. The subsequent experiments followed the temperature procedures in real-time PCR (25 °C for 1.5 min, then a stepwise increase of 1 °C every minute from 25 °C to 95 °C). During the procedures, we measured the emission intensity of FAM (520 nm) after the temperature increased. Final analysis of the data was carried out using Origin 9.0 (OriginLab Corp.).

Table S3. TTA45 stabilization by 5H-pyrazolo [4,3-c] quinoline and its derivatives as determined by FRET assays.

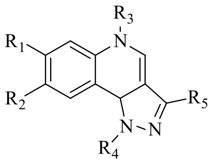

Compounds	R_1	R_2	R_3	R_4	R_5	ΔT_m/°C
27	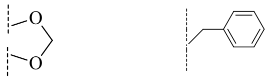			---	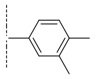	14.08 ± 2.11
BMPQ-6	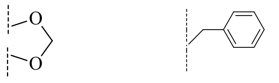			---	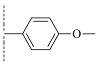	11.68 ± 0.53
28	H	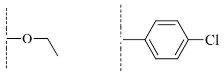		---	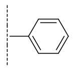	0.98 ± 0.69
29	H	CH_3	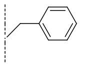	---	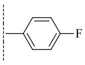	6.43 ± 0.70
BMPQ-1	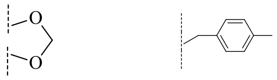			---	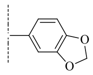	16.09 ± 1.79
BMPQ-2	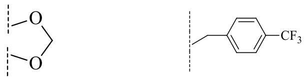			---	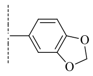	12.59 ± 0.54
BMPQ-4	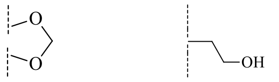			---	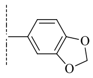	7.92 ± 0.12
30	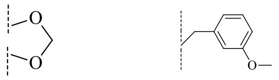			---	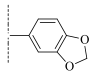	13.40 ± 1.12
31	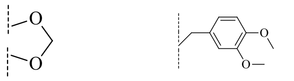			---	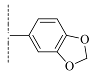	15.15 ± 0.23
32	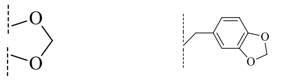			---	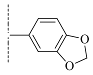	11.56 ± 0.36
33	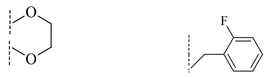			---	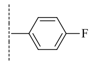	10.42 ± 1.60
BMPQ-5	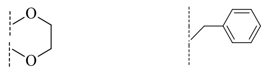			---	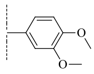	14.62 ± 0.46
34	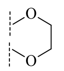		H	---	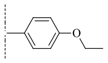	3.55 ± 1.20
35	H	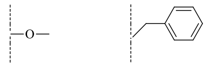		---	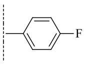	8.38 ± 1.73
BMPQ-3	H	H	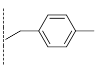	---	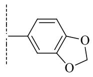	10.28 ± 0.15
36	H	H	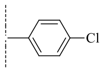	---	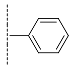	2.54 ± 0.16
37	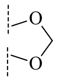		H	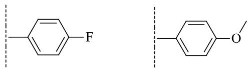		2.19 ± 0.91
38	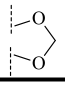		H	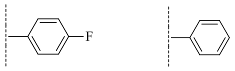		0.30 ± 0.82

Melting temperature (T_m) of 0.5 μM TTA45 with and without 20 μM compounds in solution (10 mM K_2HPO_4/KH_2PO_4, 100 mM KCl, pH 7.0) was determined by FRET procedure, and ΔT_m/°C were derived by their difference.

Table S4. Cytotoxicity of BMPQ-1 and its analogs on A549 and their ability to stabilize TTA45 and TTA

Compounds	Structures	IC_{50} /μM	ΔT_m/°C	
			TTA45	TTA
BMPQ-1	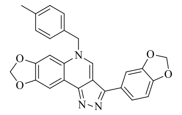	1.46 ± 0.16	14.6	8.4
BMPQ-2	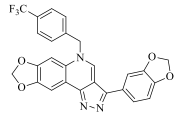	1.99 ± 0.04	7.0	2.3
BMPQ-3	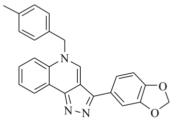	> 32	5.1	1.1
BMPQ-4	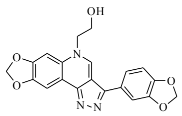	> 32	4.9	2.3
BMPQ-5	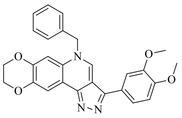	5.39 ± 0.46	4.6	4.3
BMPQ-6	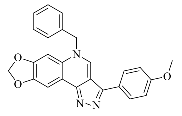	2.49 ± 0.03	3.5	1.0

[a] Melting temperature (T_m) of 5 μM DNA samples with and without 20 μM BMPQ-1 analogs in 10 mM Tris-HCl buffer (50 mM KCl, pH 7.4) was determined by circular dichroism spectroscopy, and ΔT_{m}/°C were derived by their difference.

Table S5. IC50 Values (μM) of BRACO-19, RHPS4 and BMPQ-1 against Tumor Cells.

Cell lines	IC_{50} Value (μM)		
	BRACO-19	RHPS4	BMPQ-1
HT29	14.22 ± 1.78	7.86 ± 0.73	1.40 ± 0.06
A549	6.71 ± 0.45	6.74 ± 0.99	1.46 ± 0.16
HepG2	6.25 ± 0.80	3.37 ± 0.22	2.14 ± 0.26
MGC-803	7.07 ± 0.89	4.99 ± 0.51	2.36 ± 0.48

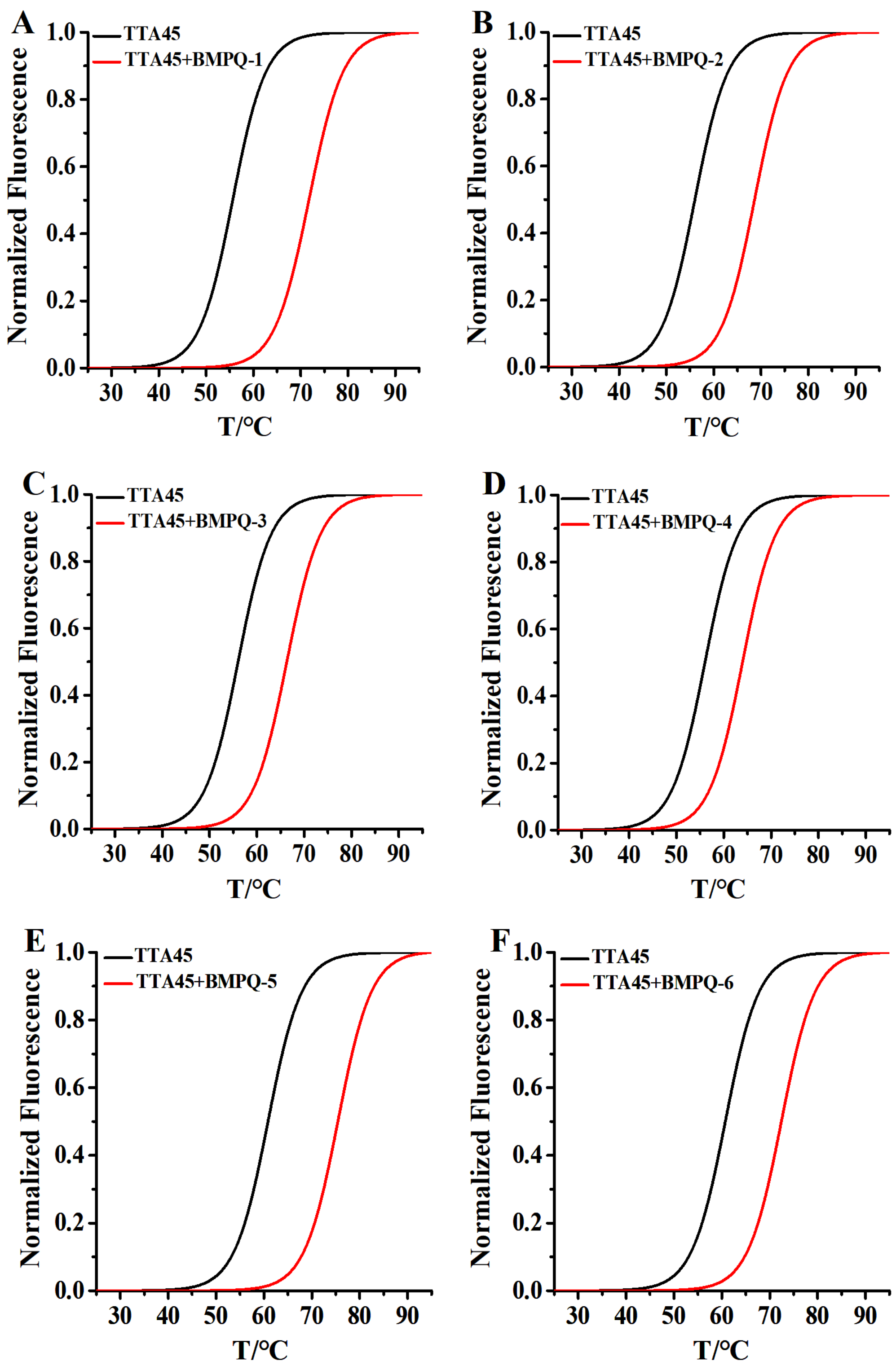

Figure S1. Normalized thermal melting curves of 0.5 μM TTA45 in the absence or presence of 20 μM BMPQ-1 and its analogs in solution with 10 mM K_2HPO_4/KH_2PO_4, 100 mM KCl, pH 7.0，All measurements were derived from FRET assays.

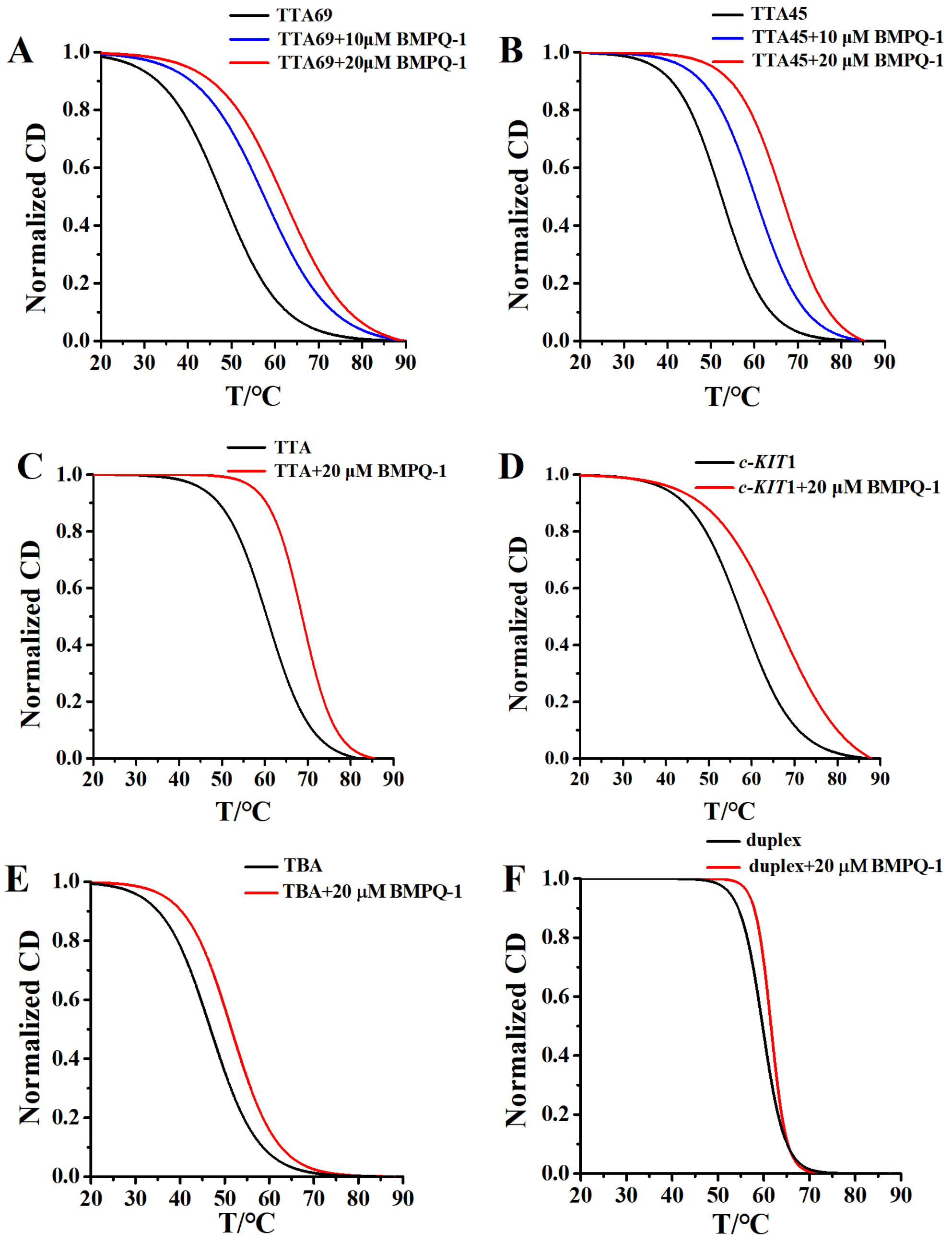

Figure S2. Normalized CD thermal melting curves of DNAs in the absence or presence of BMPQ-1 under K^+ conditions. (A) TTA69, (B) TTA45, (C) TTA, (D) *c-KIT*1, (E) TBA, (F) duplex DNA 19AT. The DNA concentration was 5 μM, and BMPQ-1 was 10 μM or 20 μM. All measurements were carried out in the buffer with 10 mM Tris-HCl (pH 7.4), 50 mM KCl.

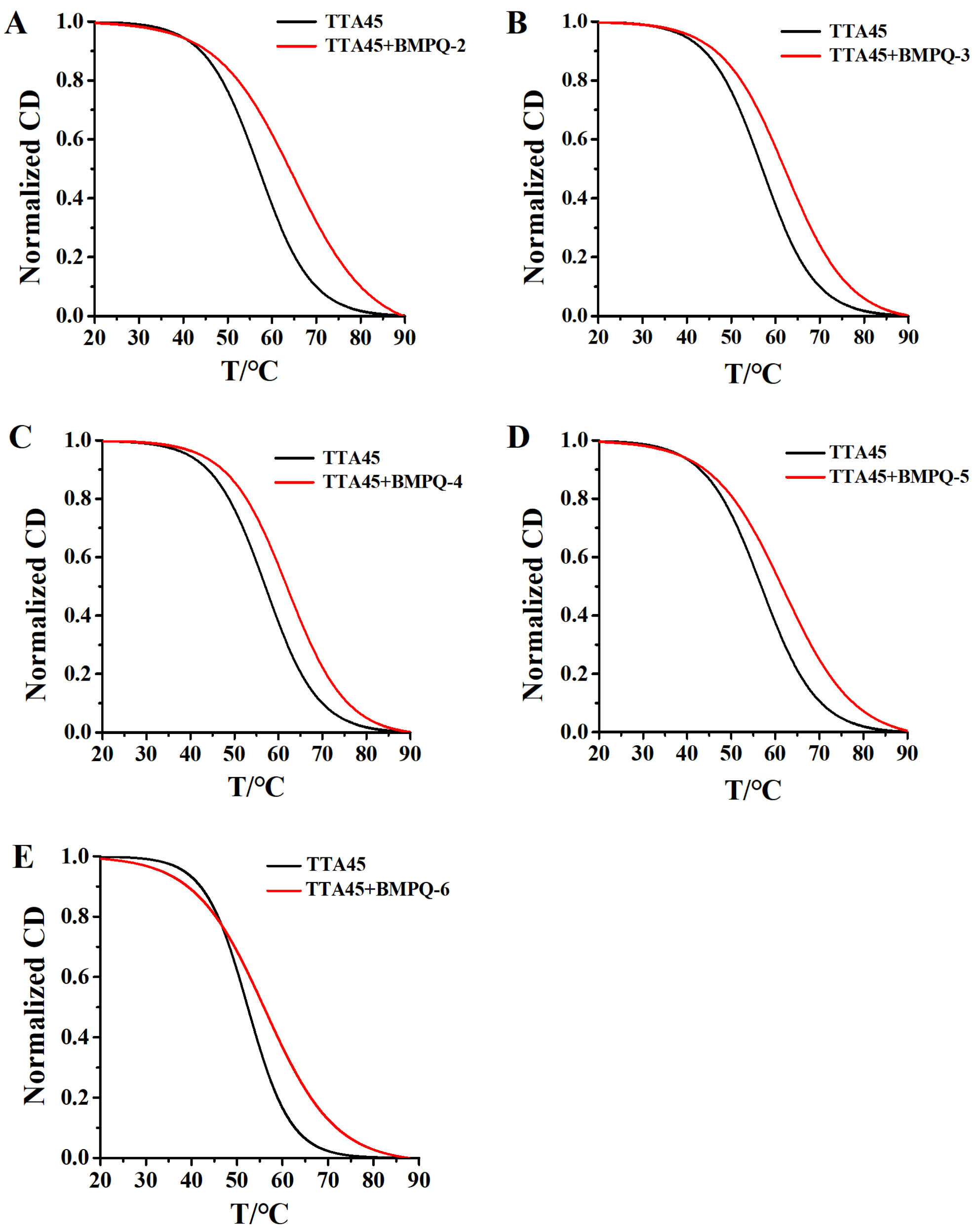

Figure S3. Normalized CD thermal melting curves of TTA45 in the absence or presence of BMPQ-1 analogs under K^+ conditions. (A) BMPQ-2, (B) BMPQ-3, (C) BMPQ-4, (D) BMPQ-5, (E) BMPQ-6. The DNA was 5 μM. The compounds were 20 μM. All measurements were carried out in the buffer with 10 mM Tris-HCl (pH 7.4), 50 mM KCl.

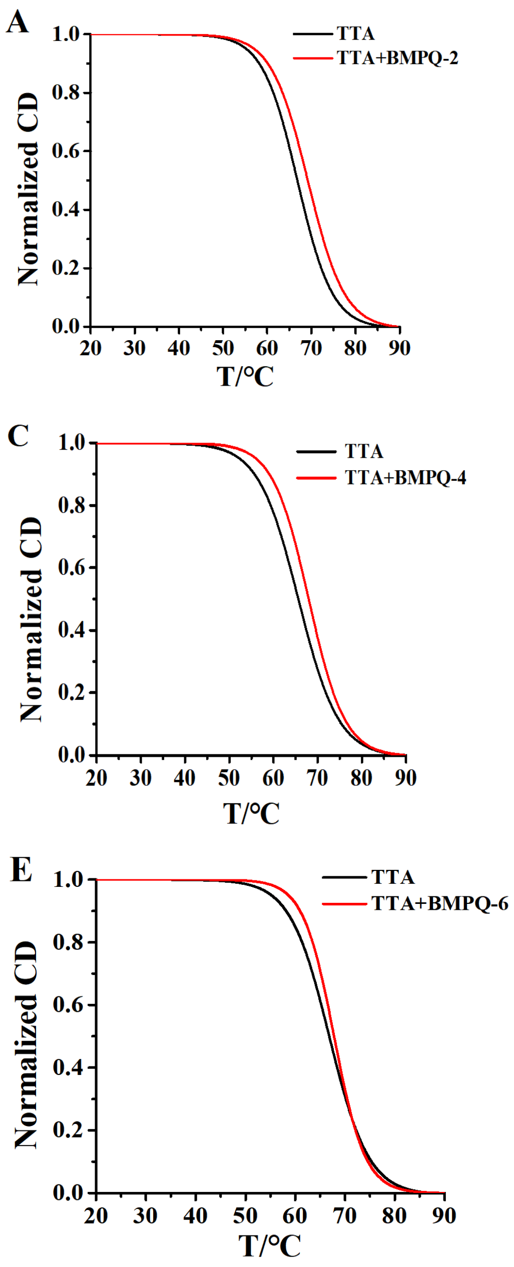

Figure S4. Normalized CD thermal melting curves of TTA in the absence or presence of BMPQ-1 analogs under K^+ conditions. (A) BMPQ-2, (B) BMPQ-3, (C) BMPQ-4, (D) BMPQ-5, (E) BMPQ-6. The DNA concentration was 5 μM. The compounds were 20 μM. All measurements were carried out in the buffer with 10 mM Tris-HCl (pH 7.4), 50 mM KCl.

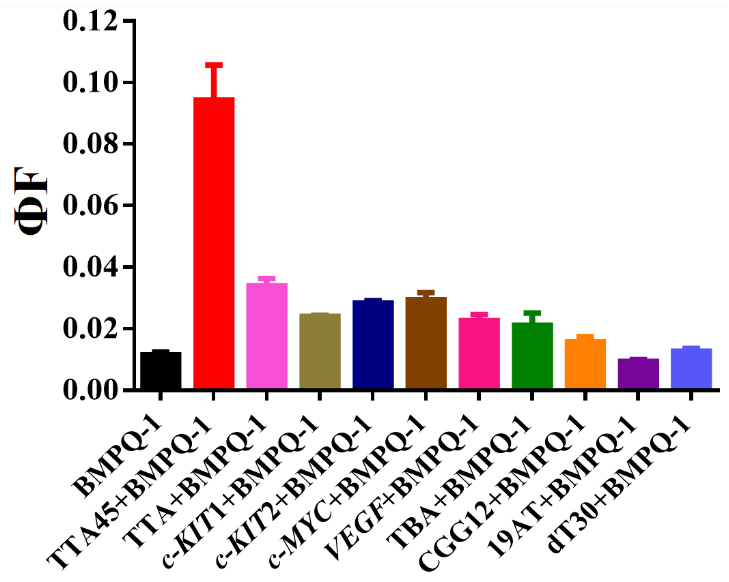

Figure S5. Fluorescence quantum yields of BMPQ-1 and BMPQ-1 with different DNA. The concentration of BMPQ-1 was 6 μM. The concentration of TTA45 was 4 μM and DNAs with other structures were 8 μM. λ_{ex} = 395 nm.

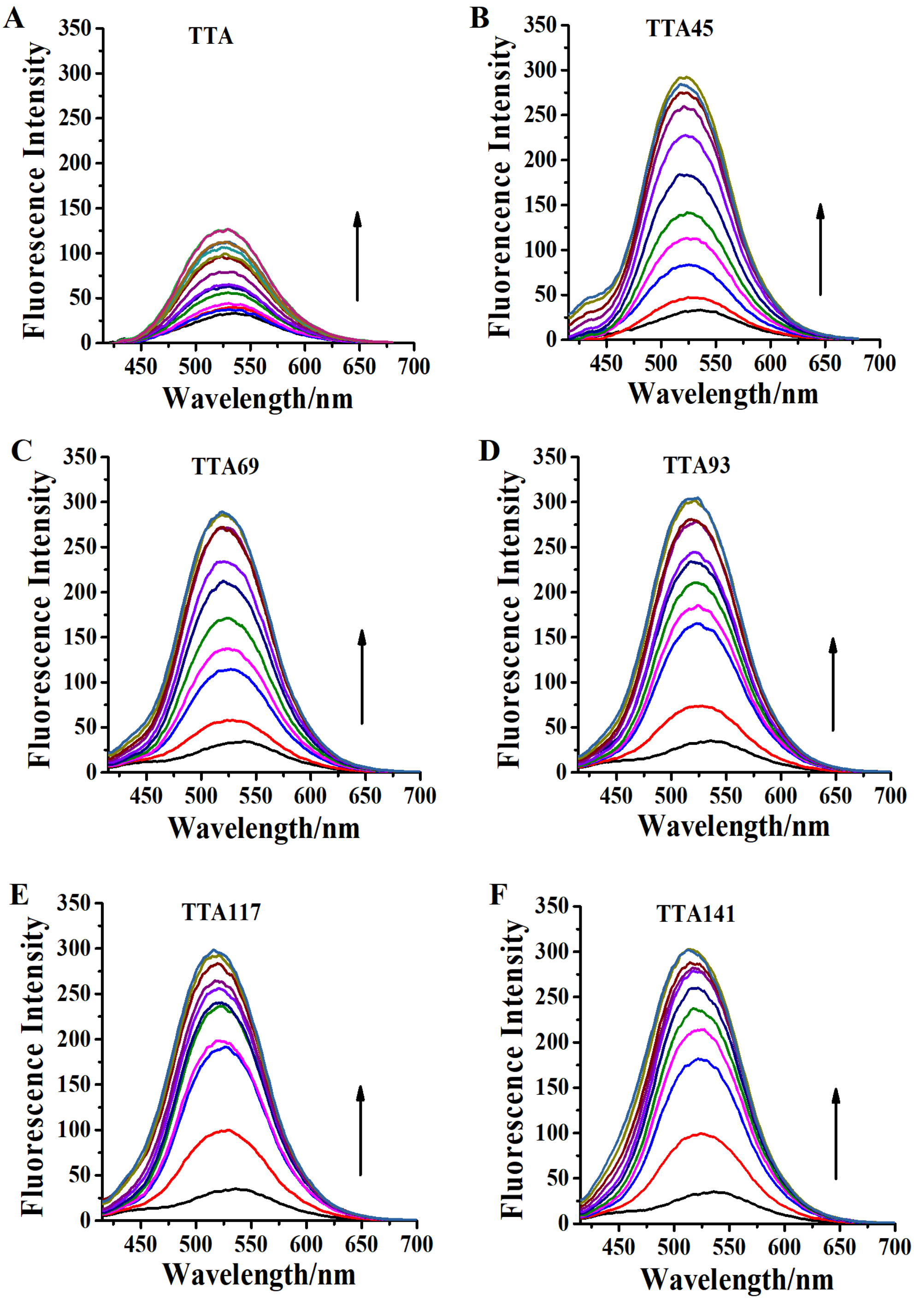

Figure S6. Fluorescence titration spectra of 3 μM BMPQ-1 against different DNAs in buffer with 10 mM K_2HPO_4/KH_2PO_4, 100 mM KCl, pH 7.0, λ_{ex} = 395 nm. (A)TTA, (B) TTA45, (C) TTA69, (D) TTA93, (E) TTA117, (F) TTA141. The concentration of TTA was 0-10 μM, and other DNAs were 0-6 μM.

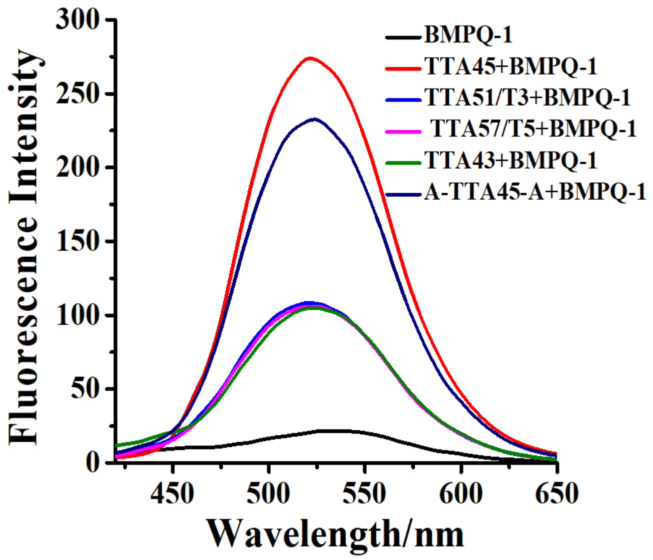

Figure S7. Fluorescence spectra of BMPQ-1 at λ_{ex} = 395 nm in the absence or presence of TTA45 and its mutants. The concentration of BMPQ-1 was 3 μM, and the concentrations of DNAs were 2 μM.

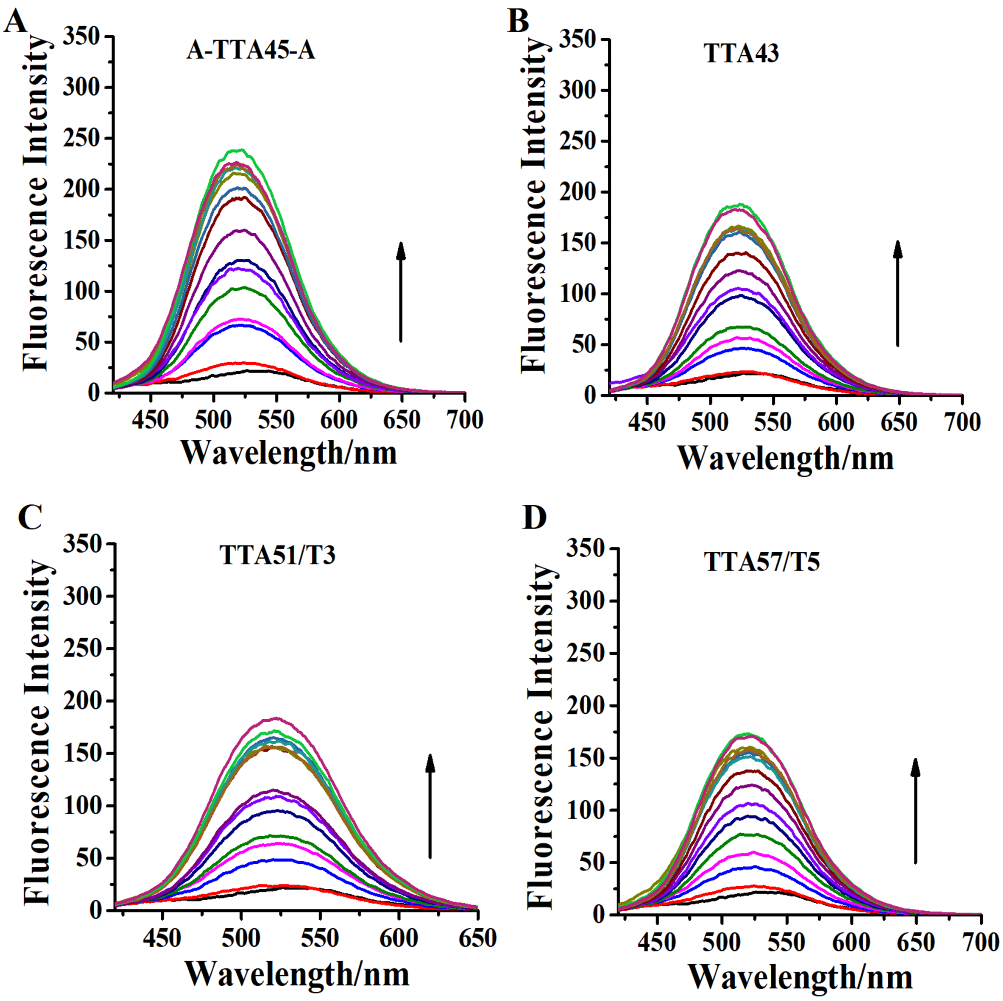

Figure S8. Fluorescence titration curves of BMPQ-1 with the stepwise addition of the multimeric G-quadruplex A-TTA45-A(A), TTA43(B), TTA51/T3(C), TTA57/T5(D). BMPQ-1 was 3 μM. The oligonucleotides were 0-10 μM in buffer with 10 mM K_2HPO_4/KH_2PO_4, 100 mM KCl, pH 7.0. λ_{ex} = 395 nm.

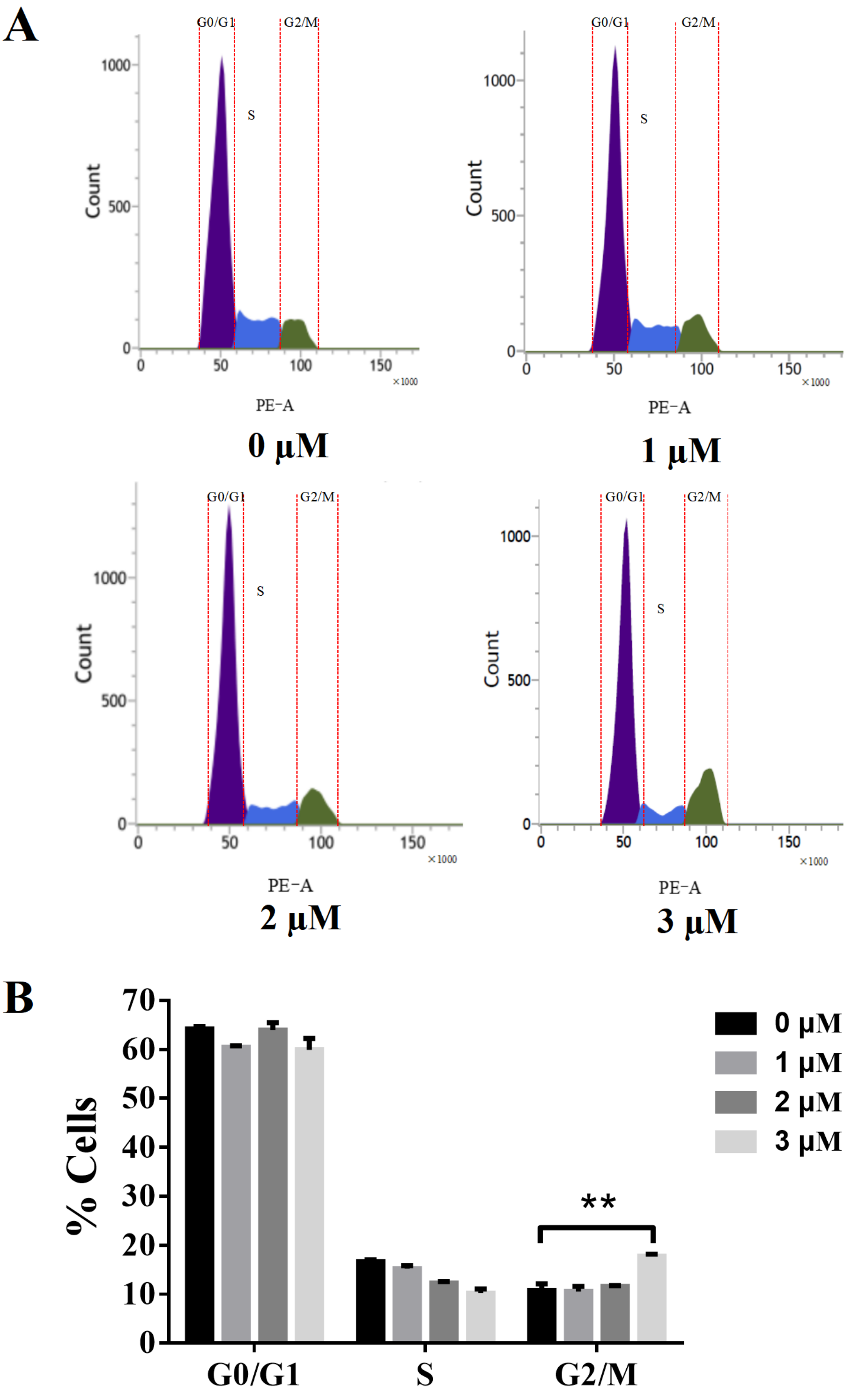

Figure S9. BMPQ-1 induced A549 cell cycle arrest. (A) The cell cycle analysis of A549 cells after 24 h treatment with BMPQ-1. The cells were collected and stained with propidium iodide (PI). (B) Percentage of A549 cells in different phases of the cell cycle. (**) $P < 0.01$, significantly different from the control.

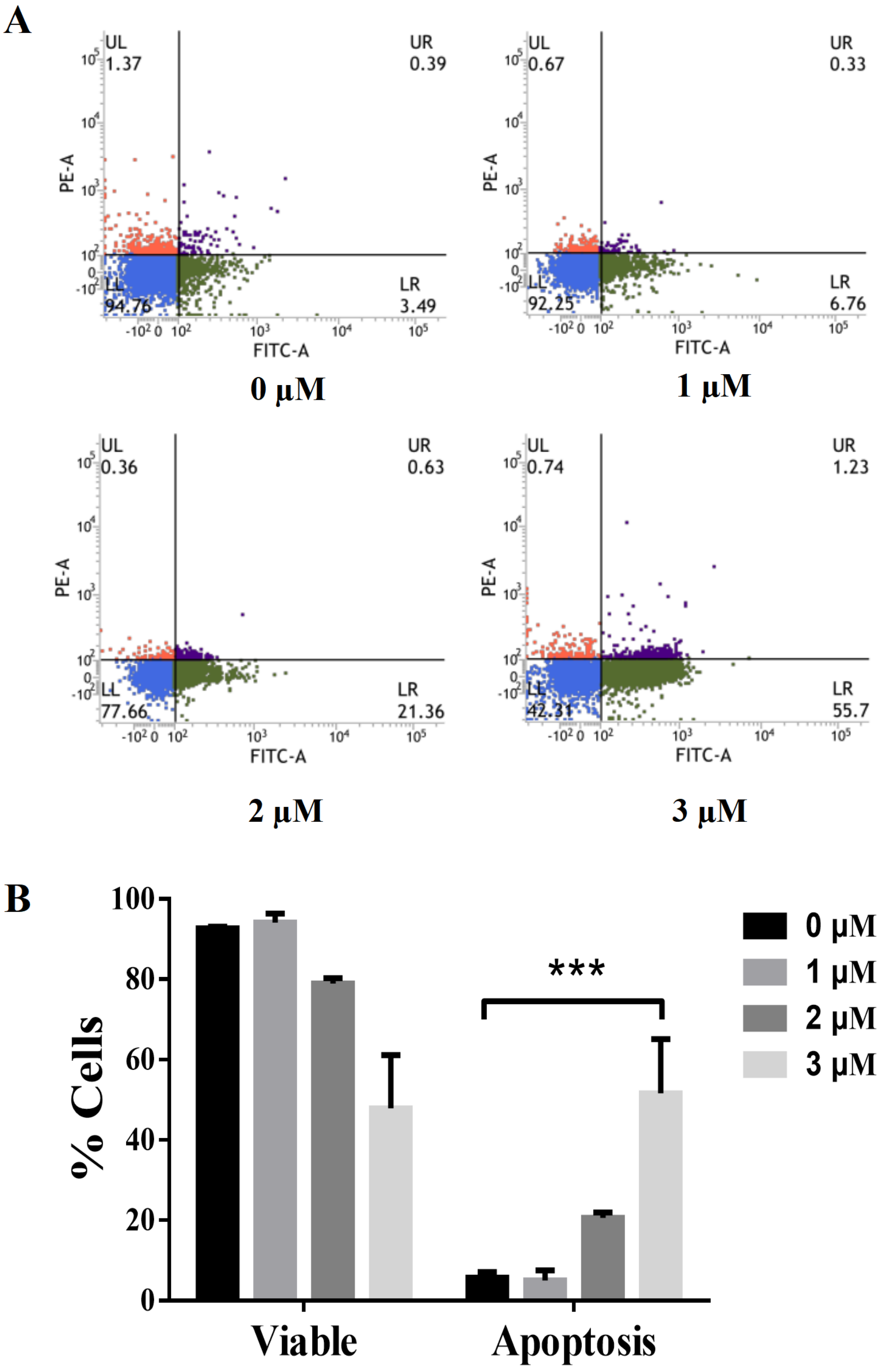

Figure S10. BMPQ-1 induced cell apoptosis of A549. (A) Apoptosis evaluation of A549 cells after 24 h treatment with various concentrations of BMPQ-1. The cells were collected and stained with Annexin V–FITC and PI. Region LL represents the viable cells; LR represents the early apoptotic cells; UR represents the late apoptotic cells; UL represents damaged cells and cell debris. (B) Percentage of apoptosis in A549 cells. (***) $P < 0.001$, significantly different from the control.

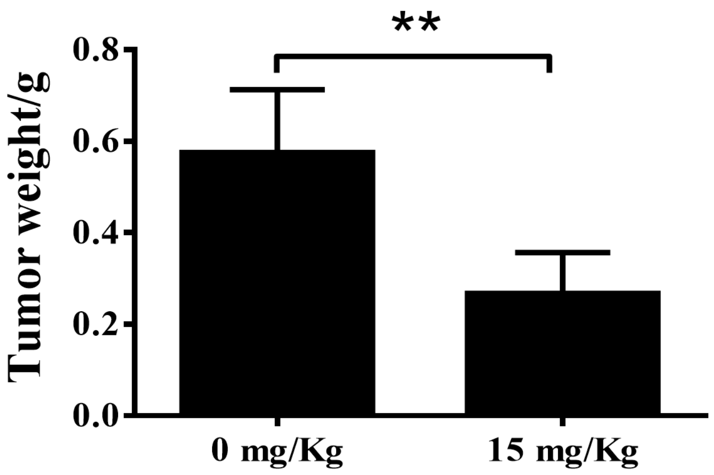

Figure S11. Weight of the excised tumors from two groups. The tumor tissues were collected after three weeks' treatment. (**) P < 0.01, significantly different from the control.

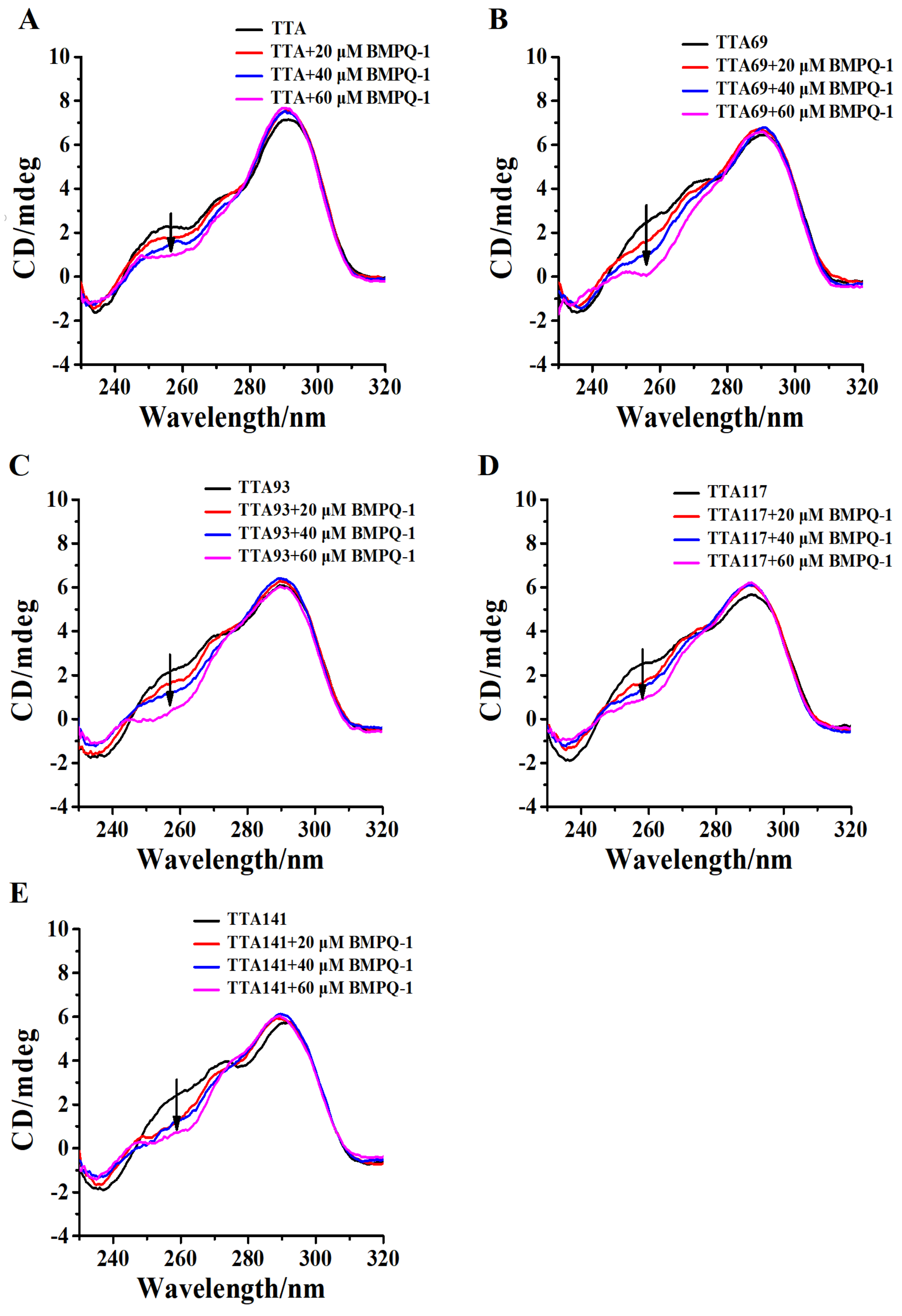

Figure S12. Effect of BMPQ-1 on the CD spectra of telomere G-quadruplexes in 10 mM Tris-HCl buffer (pH 7.4) containing 100 mM KCl. (A) TTA(G1), (B) TTA69(G3), (C) TTA93(G4), (D) TTA117 (G5), (E) TTA141 (G6). To ensure the concentration of the G-quadruplex forming units to be 20 μM, multiple G-quadruplex forming oligonucleotides were in different concentrations (20 μM TTA (G1); 6.67 μM TTA69 (G3); 5 μM TTA 93(G4); 4 μM TTA117 (G5); 3.33 μM TTA141 (G6)).

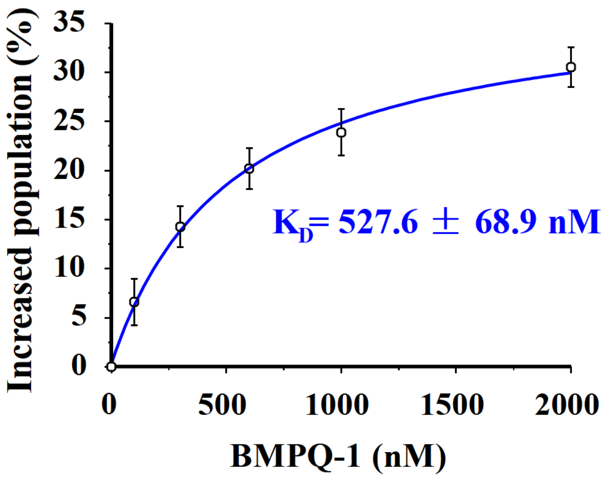

Figure S13. K_D value (527 ± 68.9 nM) was derived by fitting the population changes of high-FRET species against BMPQ-1 concentrations.

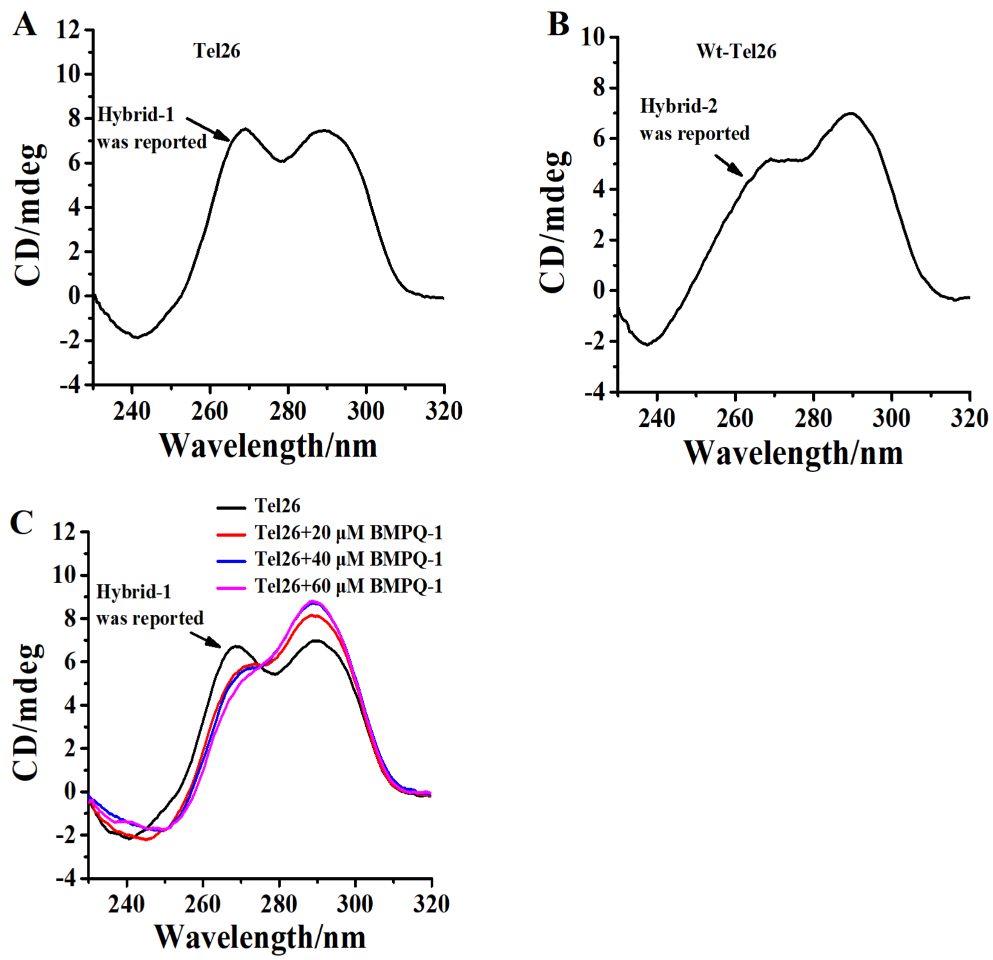

Figure S14. CD spectra of (A) Tel26 (Hybrid-1 conformation was reported), and (B) Wt-Tel26 (Hybrid-2 conformation was reported) in 10 mM Tris-HCl buffer (pH 7.4) containing 100 mM KCl. (D) Effect of BMPQ-1 on the CD spectra of Tel26 in 10 mM Tris-HCl buffer (pH 7.4) containing 100 mM KCl

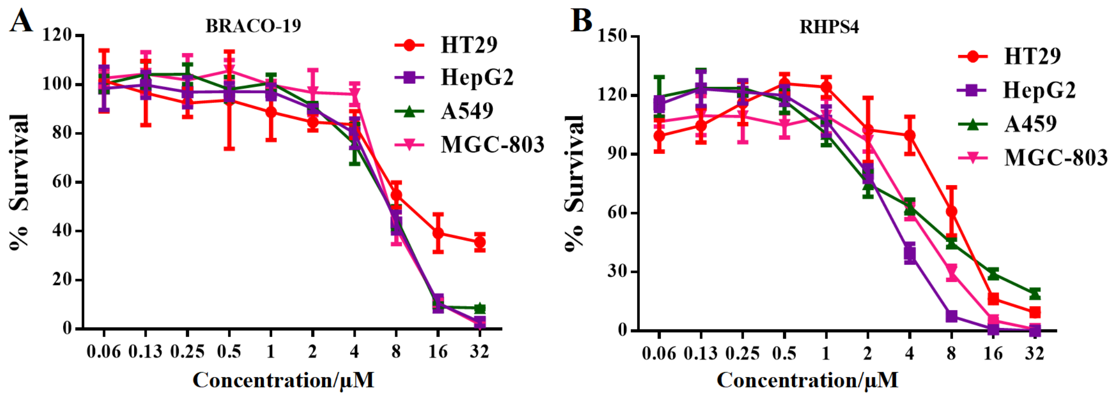

Figure S15. Cell growth inhibition curves of tumor cells and normal cells after 48 h treatment with BRACO-19 (A) and RHPS4 (B). Cell viability of the drug-treated cells were normalized to the treated with DMSO

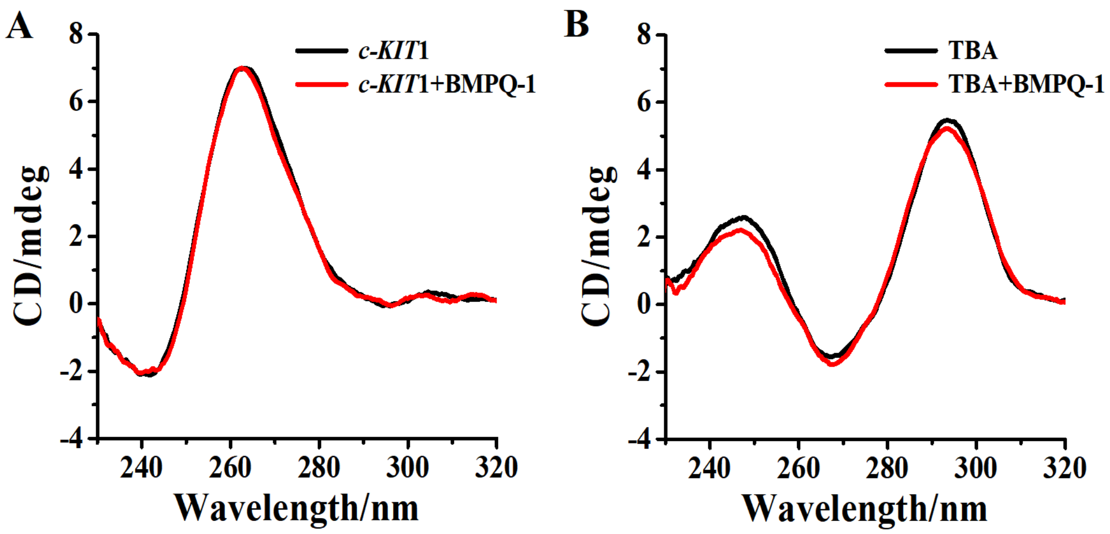

Figure S16. Effect of 60 μM BMPQ-1 on the CD spectra of 20 μM *c-KIT*1 and TBA in buffer with 10 mM Tris-HCl buffer (pH 7.4),100 mM KCl. (A) *c-KIT*1, (B) TBA.
